# Cognitive impairments correlate with increased central nervous system immune activation after allogeneic haematopoietic stem cell transplantation

**DOI:** 10.1038/s41375-023-01840-0

**Published:** 2023-02-15

**Authors:** Erik Boberg, Nadir Kadri, Daniel W. Hagey, Lilly Schwieler, Samir El Andaloussi, Sophie Erhardt, Ellen Iacobaeus, Katarina Le Blanc

**Affiliations:** 1 Department of Laboratory Medicine, Karolinska Institutet, Stockholm, Sweden; 2 Department of Haematology, Karolinska University Hospital, Stockholm, Sweden; 3 Department of Physiology and Pharmacology, Karolinska Institutet, Stockholm, Sweden; 4 Department of Clinical Neuroscience, Karolinska Institutet, Stockholm, Sweden; 5 Department of Cellular therapy and Allogeneic Stem Cell Transplantation, Karolinska University Hospital, Stockholm, Sweden

**Keywords:** Quality of life, Allotransplantation

## Abstract

Murine studies indicate that, after allogeneic haematopoietic stem cell transplantation (aHSCT), donor-derived macrophages replace damaged microglia and alloreactive T-cells invade the central nervous system (CNS). The clinical relevance of this is unknown. We assessed CNS immune surveillance and metabolic activity involved in neuronal survival, in relation to fatigue and cognitive dysfunction in 25 long-term survivors after aHSCT. Patients with cognitive dysfunction exhibited increased proportions of activated T-cells and CD16 + NK-cells in the cerebrospinal fluid (CSF). Immune cell activation was paralleled with reduced levels of anti-inflammatory factors involved in T-cell suppression (transforming growth factor-β, programmed death ligand-1), NK-cell regulation (poliovirus receptor, nectin-2), and macrophage and microglia activation (CD200, chemokine [C-X3-C motif] ligand-1). Additionally, the CSF mRNA expression pattern was associated with neuroinflammation and oxidative stress. Furthermore, proteomic, and transcriptomic studies demonstrated decreased levels of neuroprotective factors, and an upregulation of apoptosis pathway genes. The kynurenine pathway of tryptophan metabolism was activated in the CNS of all aHSCT patients, resulting in accumulation of neurotoxic and pro-inflammatory metabolites. Cognitive decline and fatigue are overlooked but frequent complications of aHSCT. This study links post-transplant CNS inflammation and neurotoxicity to our previously reported hypoactivation in the prefrontal cortex during cognitive testing, suggesting novel treatment targets.

**Figure:**
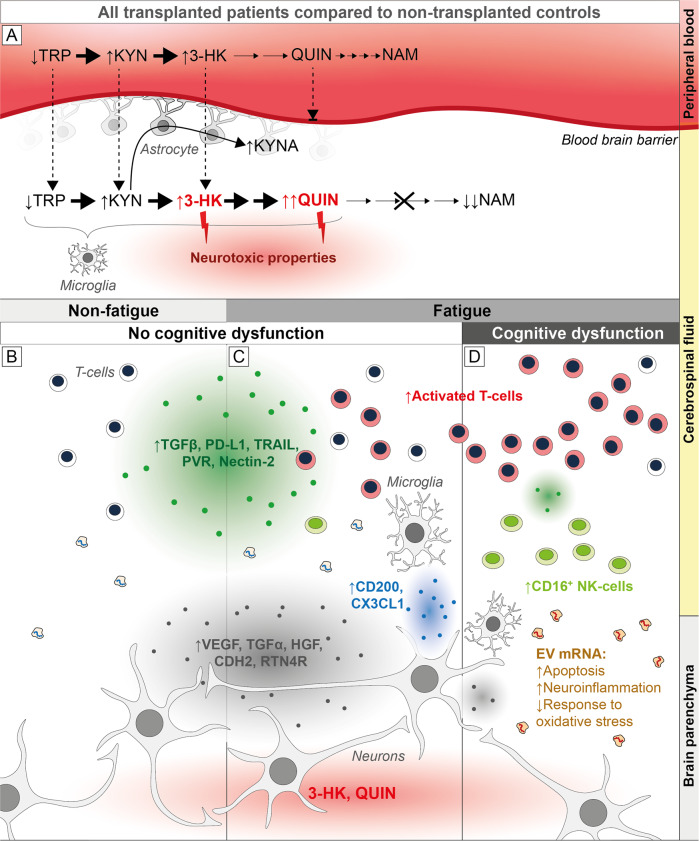
A) The kynurenine pathway of tryptophan metabolism was activated in all aHSCT patients. The levels of KYN and 3-HK, that readily cross the BBB, were increased in both plasma and CSF, while QUIN, that do not cross the BBB, was exclusively elevated in CSF, suggesting intrathecal production in microglia. QUIN and 3-HK have documented neurotoxic properties and may contribute to an underlying neurotoxic environment after aHSCT. B-D) Activated T-cells and CD16 + NK-cells were increased in the CSF of aHSCT patients with fatigue (C, D), coupled with decreased amounts of immune regulatory (green and blue dots) and neurotrophic (grey dots) proteins in the subgroup with cognitive dysfunction (D). In contrast, patients with normal cognition (B, C) had increased levels of reparative and anti-inflammatory factors, possibly counteracting the toxic environment highlighted by the increased apoptosis activity and signs of ongoing neuroinflammation observed in the mRNA sequencing analysis.

A) The kynurenine pathway of tryptophan metabolism was activated in all aHSCT patients. The levels of KYN and 3-HK, that readily cross the BBB, were increased in both plasma and CSF, while QUIN, that do not cross the BBB, was exclusively elevated in CSF, suggesting intrathecal production in microglia. QUIN and 3-HK have documented neurotoxic properties and may contribute to an underlying neurotoxic environment after aHSCT. B-D) Activated T-cells and CD16 + NK-cells were increased in the CSF of aHSCT patients with fatigue (C, D), coupled with decreased amounts of immune regulatory (green and blue dots) and neurotrophic (grey dots) proteins in the subgroup with cognitive dysfunction (D). In contrast, patients with normal cognition (B, C) had increased levels of reparative and anti-inflammatory factors, possibly counteracting the toxic environment highlighted by the increased apoptosis activity and signs of ongoing neuroinflammation observed in the mRNA sequencing analysis.

## Introduction

Allogeneic haematopoietic stem cell transplantation (aHSCT) remains the only curative option for high-risk haematological malignancies. While significant improvements have been made to decrease short-term morbidity and mortality, long-term complications still hamper treatment efficacy [1, 2]. Fatigue and cognitive dysfunction are prevalent symptoms that reduce quality of life and degree of employment for several years after treatment, even in cured patients [3, 4].

Compelling evidence suggests that the aHSCT procedure can affect brain structure and function [5, 6]: Reductions in both grey and white matter volume during the first year post transplant have been demonstrated on magnetic resonance imaging [5, 7].

Central nervous system (CNS) dysfunction after aHSCT may have multiple causes, including neurotoxic chemotherapy and infections. Recently, several lines of evidence that transplantation causes persistent neuroinflammation have emerged. Activated microglia and T cell infiltrates were reported in the brains of aHSCT treated mice and in post mortem brain tissue from acute graft-versus-host disease (GVHD) patients [8]. Sailor et al. demonstrated how busulphan causes a major loss of microglia, hence creating a permissive niche for peripheral macrophage engraftment in the brain and a permanent loss of adult neurogenesis [9]. In a murine transplant model studying cognitive behaviour, bone marrow derived macrophages and microglia were characterized as transcriptionally distinct populations [10]. Furthermore, persistence of proinflammatory macrophages and their interaction with alloreactive T-cells was identified as a distinguishing feature of the CNS of chronic GVHD (cGVHD) mice and was associated with behavioral aberrations [10].

We previously reported a descriptive cross-sectional study on patients 1–5 years after aHSCT for haematological disease [11]. A selected battery of items from the Cambridge Neuropsychological Test Automated Battery (CANTAB), combined with validated fatigue questionnaires, was used to define a distinct cohort of long-term survivors with mental fatigue and cognitive impairment. Using functional near-infrared spectroscopy (fNIRS) and electrodermal activity, we demonstrated that prefrontal cortex activity and sympathetic nervous system activity was lower in the fatigued group compared to both non-fatigued and healthy controls during cognitive testing, suggesting an underlying abnormality in neural connectivity [6].

In the present study, we hypothesized that dysfunctional neuronal activity in patients with cognitive dysfunction and fatigue after aHSCT associate with impaired CNS immune surveillance, altered metabolism, and/or a failure in neuroprotective processes. Compared to a brain biopsy, cerebrospinal fluid (CSF) can be obtained relatively non-invasively. Since CSF is drained from the brain interstitial fluid, it comprises mediators that reflect ongoing inflammation and homeostasis in the parenchyma [12]. For example, growth factors are upregulated after brain injury and transported in the CSF to promote neurogeneration and neuroprotection at injured sites [12]. While often assessed in neurological diseases, CSF inflammation has rarely been evaluated in cancer patients.

By combining analyses of immune cell subsets, protein and mRNA expression, and activation of metabolic pathways regulating inflammation and neuroprotection in the CSF, this study attempted to decipher the pathophysiology of post-transplant neurocognitive sequelae.

## Materials and methods

### Study participants and patient samples

The CSF samples were obtained from a biobank at Karolinska University Hospital (KUH), Sweden of patients sampled 1–6 years post aHSCT for haematological disease [11]. In our previous study, these patients were assessed for fatigue using the mental fatigue scale (MFS), and cognitive function using selected paradigms from the computer-based CANTAB [11].

Inclusion and exclusion criteria from the original study are summarized in supplemental Table 1. Briefly, participants were ≥18 years old, fluid in Swedish and had no history of neurologic or severe psychiatric disorders. Patients treated with intrathecal chemotherapy, CNS or total body irradiation (treatments suggested to cause CNS injury through specific mechanisms), or significant doses of psychoactive drugs were excluded. All patients were in complete haematological remission. Except for fatigue and cognitive symptoms, the included patients lacked symptoms of neurological disease, and brain MRI excluded significant structural pathology [11]. A previously published analysis of the peripheral immune compartment could not indicate differences in immune reconstitution between the groups [11]. All participants provided written consent. The study was conducted in agreement with the declaration of Helsinki and approved by the regional ethics committee in Stockholm, Sweden.

The present study included all participants (*n* = 26) with available stored CSF samples. The participants were divided into groups based on the MFS and CANTAB results. Participants with an MFS score of ≥14 were labelled as fatigued (*n* = 13). The non-fatigued group contained all other patients (*n* = 13). Cognitive dysfunction was defined as a global deficit score (GDS) ≥0.5 (*n* = 7) and no cognitive dysfunction as GDS < 0.5 (*n* = 19) [11, 13]. Cognitive dysfunction was exclusively present in the fatigued group. One patient (with fatigue and cognitive dysfunction) was excluded from the analysis as CSF microscopy and flow cytometry analysis indicated peripheral blood contamination. CSF from 12 age-, sex- and BMI-matched non-transplanted, non-inflammatory neurological disease controls (NINDC, i.e., patients with neurological symptoms without objective clinical or paraclinical findings of a neurological disease) from a biobank at KUH were included in the kynurenine pathway analysis (supplemental table 2).

### Sample collection and preparation

On average, 5.3 ml of CSF (previously suggested as adequate for flow cytometry [14]) was collected by sterile lumbar puncture. The CSF was centrifuged at 400 x *g* for 10 min at 4 °C and the supernatant was frozen in aliquots to −80 °C. The cell pellets were resuspended in pooled human AB plasma supplemented with 10% (v/v) dimethyl sulfoxide (DMSO; Sigma-Aldrich Sweden AB. Stockholm, Sweden) and stored in liquid nitrogen.

### CSF protein profiling

A total of 274 proteins were measured in the CSF using the Olink® Target 96 Metabolism, Neurology and Inflammation panels (Olink Proteomics AB, Uppsala, Sweden), employing a Proximity Extension Assay (PEA) technology (Supplemental table 3) [15]. The final assay read-out is presented in Normalized Protein eXpression (NPX) values, which is an arbitrary unit on a log2-scale where a high value corresponds to a higher protein expression. For details, refer to the supplemental methods.

### mRNA sequencing

CSF supernatants were centrifuged at 2000 x *g* for 10 min to remove non-extracellular vesicle (EV) particles and debris. Subsequently, RNA was extracted using TRI reagent (Sigma-Aldrich, USA) and precipitated according to Hagey et al., 2021 [16]. cDNA was produced using the Smart-seq2 RNA-sequencing protocol [17] and 50 bp single end reads were sequenced on an Illumina HiSeq 3000 (Illumina, USA). Sequencing was performed twice for each sample. Reads were then mapped to the ENSEMBL human transcriptome GRCh37 using Tophat 2.1.1. For details, refer to the supplemental methods.

### Flow cytometry analysis

As CSF cells are scarce, we opted to analyse the full samples, without prior counting. CSF cells were thawed and washed twice with RPMI media supplemented with 10% (v/v) fetal calf serum (ThermoFisher Scientific, Stockholm, Sweden). Cell pellets were first stained with 1ul of anti-CCR7 for 30 min at 37 °C. Subsequently, they were incubated with the additional antibodies for another 20 min at 4 °C. Supplemental table 4 lists all antibodies and concentrations used. LIVE/DEAD™ fixable aqua dead cell stain was used to assess cell viability (ThermoFisher Scientific). Cells were run on a FACSymphony™ (Becton Dickinson) and data were analyzed using FlowJo X software. After removal of debris, an average of 1662 cells were acquired per sample.

### Ultra-performance liquid chromatography (UPLC-MS/MS)

Tryptophan, kynurenine, kynurenic acid (KYNA), quinolinic acid (QUIN), picolinic acid (PIC), 3-hydroxykynurenic acid (3-HK), nicotinamide (NAM) and nicotinic acid were quantified in CSF and plasma by UPLC-MS/MS system using a Xevo TQ-XS triple-quadrupole mass spectrometer (Waters, Manchester, UK) [18]. The samples were analysed in duplicates with <5% intra-assay variation. All metabolites measured in CSF and plasma samples were detected in higher concentrations than the lowest detection level in plasma. For a detailed description, refer to the Supplemental methods.

### Statistical analysis

Unless otherwise specified, normality was determined using the Shapiro-Wilk test. Normally distributed populations (*p* < 0.05) were compared using ANOVA with post-hoc Tukey’s honest significant difference test, or student’s *T*-test. Non-normal populations were compared using Kruskal–Wallis’ test with post-hoc Dunn’s test, or Wilcoxon rank sum test. Statistical analyses were performed using R version 4.1 [19]. Principal component analysis was performed using the prcomp function in R.

#### Proteomic data

Potential outliers were examined by plotting interquartile range vs sample median. No outliers were found. Proteins that were below the limit of detection in ≥50% of the samples (*n* = 55) were excluded from further analysis. NPX values from the proteomic analysis were then compared using t-tests. The analysis was performed using the OlinkAnalyze package for R version 4.1 [20]. Before heatmap visualization, the NPX values were scaled to a mean of 0 and a standard deviation of 1. Protein interactions were evaluated using the STRING database [21].

#### mRNA expression data

Differential expression was assessed using the Deseq2 package to compare duplicate samples in R [22]. Up- and downregulated genes with an adjusted *p*-value < 0.05 were separately analysed using Panther Gene Ontology Analysis (panther.org), with all genes passing this threshold used as a control group. Gene ontology term fold enrichment for up- or downregulated genes were then divided by the fold enrichment in the control group to produce the relative term fold enrichment.

#### Correlations

CSF immune cell subsets and kynurenine pathway metabolites were correlated to protein levels using simple linear regression. One model was constructed for each correlation with either a kynurenine metabolite or a cell subset as dependent variable, and a protein as the independent variable.

#### Multiple comparison correction

The proteomic, mRNA expression and correlation analyses were adjusted for multiple comparisons using the Benjamini-Hochberg method. As this was an exploratory, hypothesis-generating study, further adjustments were not made.

## Results

Demographic and clinical characteristics of the study cohort are summarized in Table 1. Apart from BMI, no patient- or transplant-related variables significantly differentiated between non-fatigue and fatigue, or between patients with and without cognitive dysfunction.

**Table 1:** Characteristics of the study population.

	F (*n* = 12)	Non-F (*n* = 13)	P	CD (*n* = 6)	Non-CD (*n* = 19)	*P*
*Age at inclusion (years):* median (range)	56.5 (22–69)	60 (23–73)	0.49^g^	55.5 (23–69)	57 (22–73)	0.92^h^
*Sex (M): n* (%)	4 (33)	7 (54)	0.43^i^	2 (33.3)	9 (47.4)	0.66^i^
*Time from transplant to CSF sampling (months):* mean (sd)	37.6 (16.4)	34.6 (16.9)	0.66^g^	39.5 (18.6)	34.9 (16.0)	0.63^h^
*Donor type: n* (%)						
MUD	9 (75)	8 (61.5)	1^i^	5 (83.3)	12 (63.2)	0.72^i^
SIB	3 (25)	4 (30.8)		1 (16.7)	6 (31.6)	
Haplo	0 (0)	1 (7.7)		0 (0)	1 (5.3)	
*Underlying disease: n* (%)						
AML	4 (33)	10 (76.9)	0.13^i^	2 (33.3)	12	0.22^i^
CML	1 (8.3)	0 (0)		1 (16.7)	0 (0)	
MDS	2 (16.7)	2 (15.4)		1 (16.7)	3 (15.8)	
MDS/AML	1 (8.3)	0 (0)		0 (0)	1 (5.3)	
PMF	1 (8.3)	0 (0)		0 (0)	1 (5.3)	
PMF/AML	0 (0)	1 (7.7)		0 (0)	1 (5.3)	
Myeloma	1 (8.3)	0 (0)		1 (16.7)	0 (0)	
CLL	1 (8.3)	0 (0)		0 (0)	1 (5.3)	
Sickle cell anemia	1 (8.3)	0 (0)		1 (16.7)	0 (0)	
*Conditioning regimen drugs: n* (%)						
Busulfan	7 (58.3)	10 (76.9)	0.41^i^	3 (50)	14 (73.7)	0.34^i^
Cyclophosphamide	3 (25)	2 (15.4)	0.64^i^	1 (16.7)	4 (21.1)	1^i^
Fludarabine	9 (75)	11 (84.6)	0.64^i^	5 (83.3)	15 (78.9)	1^i^
Treosulfan	5 (41.7)	3 (23.1)	0.41^i^	3 (50)	5 (26.3)	0.34^i^
Thiotepa	1 (8.3)	0 (0)	0.48^i^	1 (16.7)	0 (0)	0.24^i^
*Immune reconstitution*						
*Days from transplant to neutrophils* *>* *0.5* *×* *10*^*9*^*/L (days):* mean (sd)	16.8 (4.29)	16.2 (2.45)	0.83^h^	17.5 (5.86)	16.2 (2.32)	0.61^g^
*CMV and EBV: n* (%)						
*CMV mismatch*^*a*^	5 (41.7)	3 (23.1)	0.41^i^	3 (50)	5 (26.3)	0.34^g^
*EBV mismatch*^*b*^	0 (0)	1 (7.7)	1^i^	0 (0)	1 (5.3)	1^g^
*CMV reactivation*^*c*^	5 (41.7)	4 (30.8)	0.69^i^	3 (50)	6 (31.6)	0.63^g^
*EBV reactivation*^*d*^	1 (8.3)	1 (7.7)	1^i^	1 (16.7)	1 (5.3)	0.43^g^
*GvHD prophylaxis: n* (%)						
*ATG*	10 (83.3)	8 (61.5)	0.38^i^	6 (100)	12 (63.2)	0.14^g^
*Ciclosporin*	8 (66.7)	10 (76.9)	0.57^i^	3 (50)	15 (78.9)	0.055^g^
*Tacrolimus*	3 (25)	1 (7.7)		3 (50)	1 (5.3)	
*Tacrolimus* *+* *Sirolimus*	1 (8.3)	2 (15.4)		0 (0)	3 (15.8)	
*GvHD: n* (%)						
*Acute*^*e*^	7 (58.3)	7 (53.8)	1^i^	2 (33.3)	12 (63.2)	0.35^g^
*Chronic*^*f*^	4 (33.3)	4 (30.8)	1^i^	0 (0)	8 (42.1)	0.13^g^

^a^Either donor or recipient (but not both) had a positive serology for CMV before aHSCT.

^b^Either donor or recipient (but not both) had a positive serology for EBV before aHSCT.

^c^Defined as CMV DNA > 1000 copies/ml. ^d^Defined as EBV DNA > 1000 copies/ml.

^e^Previous acute GvHD requiring steroid treatment. ^f^Previous/current cGvHD.

^g^Students *t*-test (continuous data with parametric distribution according to the Shapiro–Wilk test).

^h^Wilcoxon Rank Sum Test (continuous data with non-parametric distribution according to the Shapiro–Wilk test).

^i^Fisher’s exact test (categorical data). *MUD* Matched Unrelated Donor, *SIB* Matched Sibling Donor, *Haplo* Haploidentical donor, *AML* Acute Myeloid Leukemia, *CML* Chronic Myeliod Leukemia, *MDS* Myelodysplastic Syndrome, *PMF* Primary Myelofibrosis, *CLL* Chronic Lymethylphenidateocytic Leukemia, *CMV* Cytomegalovirus, *EBV* Epstein Barr Virus, *GvHD* Graft versus Host Disease, *ATG* Anti Thymocyte Globulin, *SSRI* Selective Serotonin Reuptake Inhibitor.

### Patients with fatigue had increased levels of activated T-cells in the CSF and patients with cognitive dysfunction also exhibited elevated levels of CD16+NK-cells

**Fig. 1:**
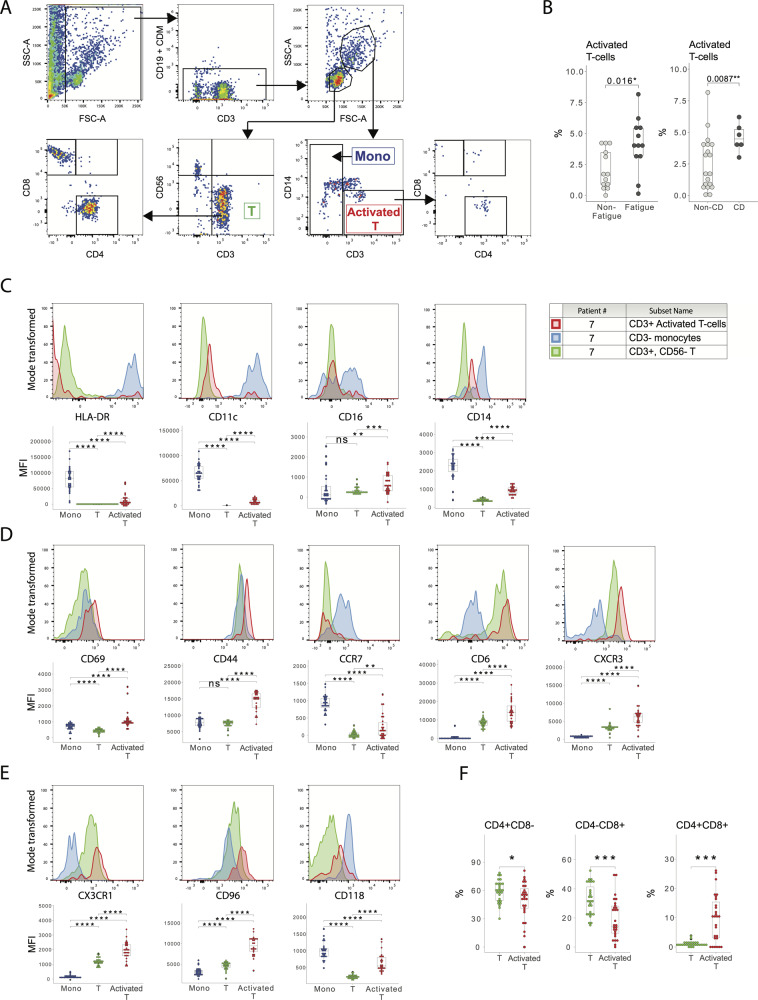
Proportion and activation of T-cells in the CSF of patients with fatigue and cognitive dysfunction. **A** Gating strategy for the flow cytometry analysis on CSF. In the FSChiSSChi gate, a population of CD3 + CD14low cells were identified and labelled as “Activated T”. Monocytes were defined as the CD3- population in the same gate. In the FSClowSSClow gate, regular T-cells were defined as being CD3 + CD56-. **B** The proportion of “Activated T-cells” were higher in patients with fatigued and cognitive dysfunction, compared to non-fatigued patients, and to those without cognitive dysfunction, respectively. **C**–**E** The “Activated T-cells” expressed low levels of monocyte markers. The expression of markers for T-cell activation and the chemokine receptor CX3CR1 was higher compared to regular T-cells. Both CD6 and CD96 (whose ligands; CD318 and CD112, respectively, were underexpressed in patients with cognitive dysfunction) were both over-expressed on activated T-cells. The soluble form of LIF-R was under-expressed in patients with cognitive dysfunction, while increased expression of the membrane bound form was detected on activated T-cells. The activated T-cells were to a greater extent double positive for CD4 and CD8. CD Cognitive dysfunction, CSF Cerebrospinal fluid. *p < 0.05, **p < 0.01, ***p < 0.005, ****p < 0.0001.

We investigated the existence of allo-immune responses in the CNS of patients with cognitive dysfunction and fatigue by using high-dimensional flow cytometry. Within the FSChiSSChi cell subset (Fig. 1A), a significantly increased level of CD3^+^CD14^low^ T-cells was detected in both patients with fatigue and cognitive dysfunction, compared to patients without fatigue or cognitive dysfunction (Fig. 1B), suggesting the existence of an expanded T cell population [23]. Further analysis revealed that the CD3^+^CD14^low^ T-cells also expressed higher levels of T-cell activation markers CD69, CD44, C-C chemokine receptor-7 (CCR7), CD6 and C-X-C Motif Chemokine Receptor-3 (CXCR3), compared to T-cells in the FSC^low^SSC^low^ gate (Fig. 1D). Moreover, they expressed significantly higher levels of CD14, CD16, CD11c and HLA-DR, compared to T-cells in the FSC^low^SSC^low^ gate, although the levels remained significantly lower than in monocytes (Fig. 1C). Consequently, the FSC^hi^SSC^hi^CD3^+^CD14^low^ cells were labelled as “activated T-cells” in the present study.

Compared to T-cells in the FSC^low^SSC^low^ gate, a significantly higher proportion of the activated T-cells were double positive for CD4 and CD8 (Fig. 1C). The increased proportion of activated T-cells was not associated with alterations of antigen presenting cells in the CSF (monocytes and dendritic cells; supplemental Fig. 1 and Supplemental tables 5–6).

**Fig. 2:**
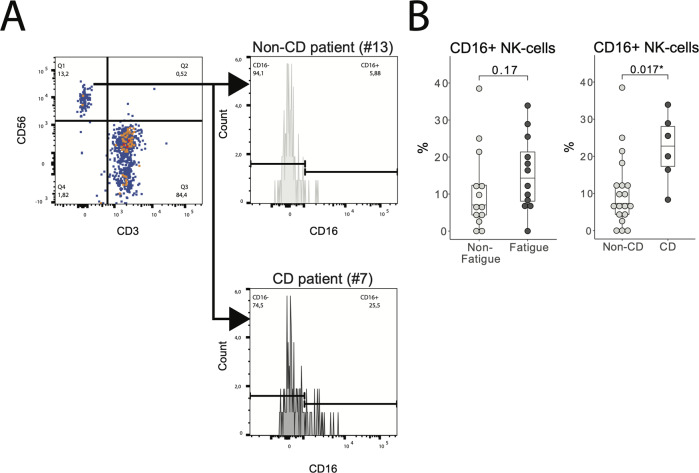
CD16 + NK-cells in patients with and without cognitive dysfunction. **A** Gating strategy for NK-cells. In the FSClowSSClow gate (see Fig. 1), NK-cells were defined as CD3-CD56+. CD16+and CD16- NK-cells were separated as shown. **B** The proportion of CD16 + NK-cells was higher in the patients with compared to without cognitive dysfunction. No difference was observed between fatigued and non-fatigued patients. CD Cognitive dysfunction. *p < 0.05.

NK-cells were defined as CD56^+^CD3^-^ cells in the FSC^low^SSC^low^ gate (Fig. 2A). No difference was found between fatigued and non-fatigued patients (Fig. 2B, left panel). However, patients with cognitive dysfunction had a higher proportion of CD16^+^ NK-cells, compared to those with normal cognition (Fig. 2B, right panel).

Combined, the flow analysis demonstrated activation of both innate and adaptive immune responses in patients with fatigue and cognitive dysfunction.

### Increased T- and NK-cell activity correlated with decreased immune regulatory and neurotrophic proteins in the CSF of patients with cognitive dysfunction

**Fig. 3:**
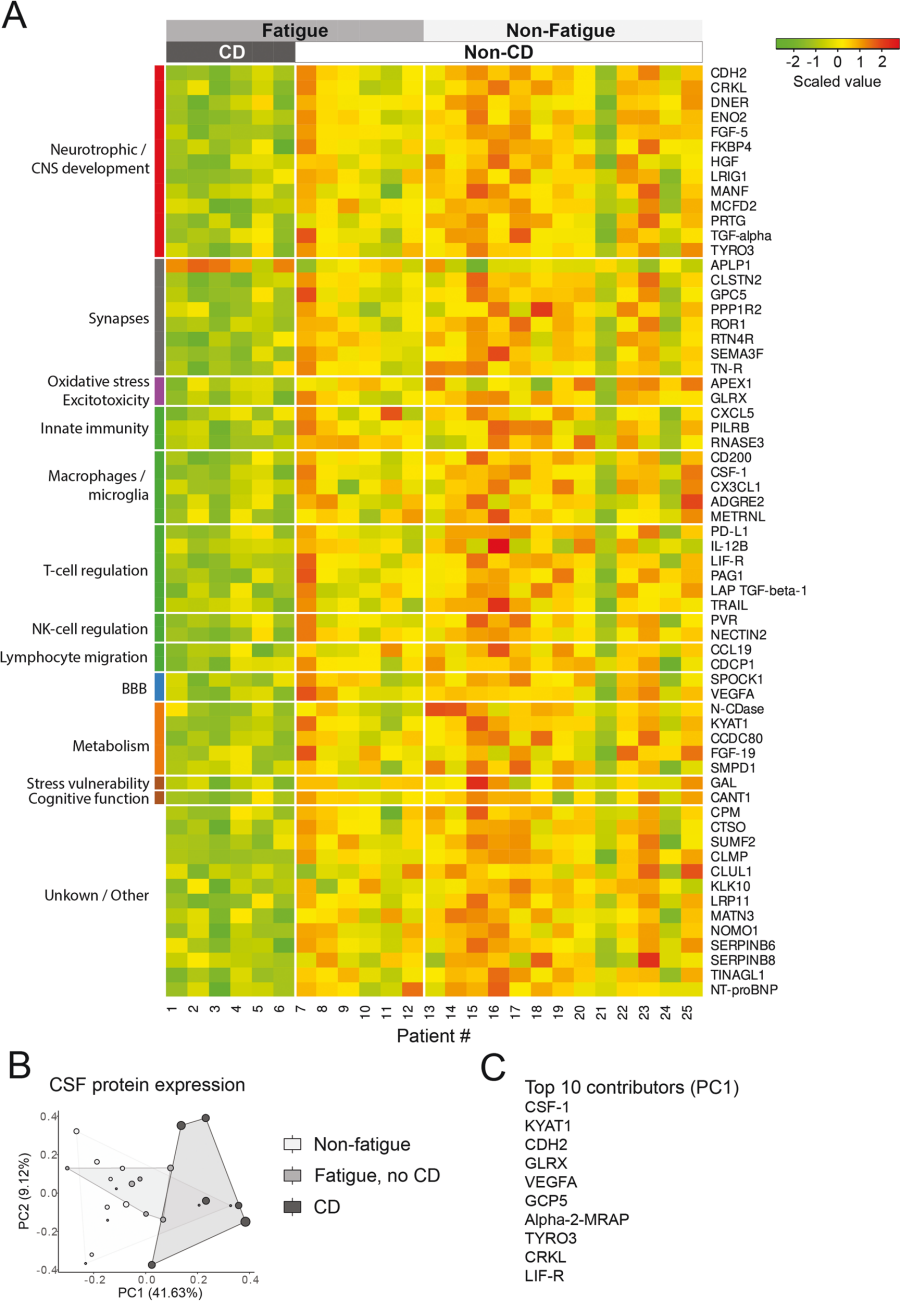
Proteomic analysis of CSF from patients with or without cognitive dysfunction and fatigue. **A** A heatmap of the 63 differentially expressed proteins that were significantly downregulated in the patients with cognitive dysfunction, compared to those without. The proteins are categorized according to a literature search (Supplemental table 7). **B** PCA reveals that patients with cognitive dysfunction segregate from the non-fatigued controls, while the fatigued patients without cognitive dysfunction do not. **C** The top 10 contributors to PC1. CD Cognitive Dysfunction, CNS Central Nervous System, BBB Blood-Brain Barrier, PCA Principal Component Analysis.

Among the 276 analysed proteins in the CSF, 219 were detected and 63 were differentially expressed between patients with and without cognitive dysfunction (Fig. 3A). All differentially expressed proteins were downregulated in the cognitive dysfunction group with the exception of Amyloid-like protein-1 (APLP1), a synaptic protein suggested as a biomarker for Alzheimer’s disease, that was significantly increased [24]. The distinct protein expression was evident in a principal component analysis that distinguished patients with cognitive dysfunction from the other patient groups (Fig. 3B, C).

When comparing patients with and without fatigue, no proteins were significantly different.

All proteins that were expressed at significantly different levels in patients with and without cognitive dysfunction were functionally annotated based on information from databases and literature concerning their role in CNS and immune system pathology (Fig. 3A, supplemental Fig. 2A, Supplemental table 7). Accordingly, patients with cognitive dysfunction had reduced levels of neurotrophic growth factors such as vascular endothelial growth factor (VEGF), hepatocyte growth factor (HGF), transforming growth factor alpha (TGFα), TGF beta-1 (TGFβ1), and cadherin-2 (CDH2) as well as proteins important for synapse plasticity including ROR1 and calsyntenin-2 [25–31]. In addition, negative regulators of both innate and adaptive immunity in the CNS were lower in patients with cognitive dysfunction compared to those without. Notably, CD200, colony stimulating factor-1 (CSF1) and chemokine (C-X3-C motif) ligand-1 (CX3CL1), all involved in the regulation of macrophages and microglia as well as the T-cell regulatory proteins programmed death ligand-1 (PD-L1), leukemia inhibitory factor receptor (LIF-R), TNF-related apoptosis-inducing ligand (TRAIL) and TGFβ1, and poliovirus receptor (PVR) as well as Nectin-2 were decreased [32–38]. STRING analysis revealed highly confident interactions between downregulated neurotrophic and immune regulatory proteins (Supplemental Fig. 2A) [21].

### CSF mRNA expression corroborated cell and protein analysis with findings of proinflammatory activity, reduced oxidative stress and apoptosis regulation in patients with cognitive dysfunction

Extracellular vesicles contain the majority of extracellular mRNA in CSF and play important physiological roles in CNS transcellular communication [39, 40]. As such, they are proposed to impact numerous pathophysiological pathways in neurodegenerative diseases [41].

**Fig. 4:**
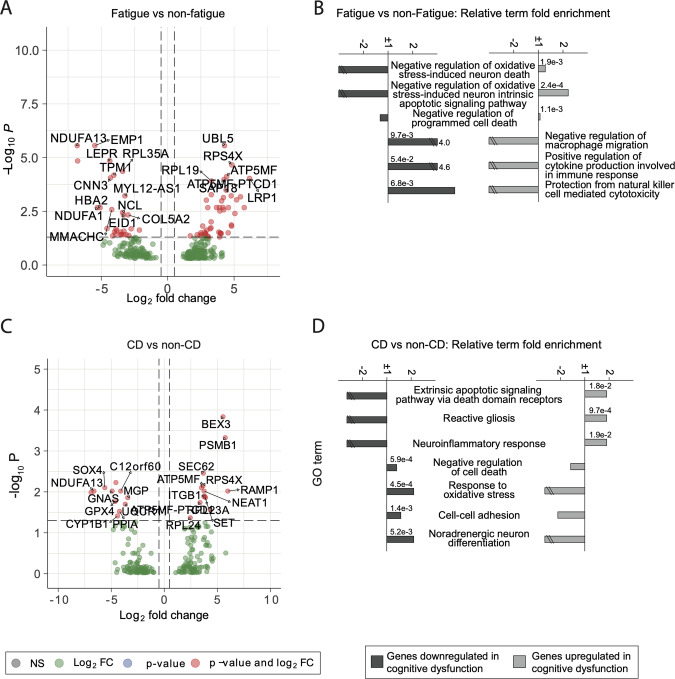
mRNA expression in cell-free CSF from patients with and without fatigue and cognitive dysfunction. **A**, **C** Volcano plots of mRNA expression in CSF EVs. A negative Log2-fold change indicates decreased expression in patients with cognitive dysfunction. **B**, **D** Selected GO-terms from the results of the Panther Gene Ontology Analysis. The relative fold enrichment among both up- and downregulated mRNA are displayed. The terms that were enriched among the downregulated genes were negatively enriched among the upregulated genes and vice versa. The *P*-values prior to FDR adjustment are displayed. CD Cognitive dysfunction.

Sequencing of CSF extracellular mRNA in all patients with fatigue revealed 76 genes differentially expressed compared to patients without fatigue (Fig. 4A). GO-term analysis suggested reduced apoptosis and autophagy as well as decreased production of cytokines involved in immune responses and immune cell migration (Fig. 4B).

Focusing on cognition, 24 genes significantly differed between patients with and without cognitive dysfunction (Fig. 4C). GO-terms from the downregulated genes included processes important for cell-cell adhesion, integral to axon guidance and synaptic plasticity [42], as well as responses to oxidative stress, noradrenergic neuron differentiation and negative regulation of cell death (Fig. 4D). In contrast, GO terms from the upregulated gene set contained neuroinflammatory response genes, reactive gliosis and the extrinsic apoptotic signalling pathway via death domain receptors. All selected GO terms are listed in supplemental Fig. 3.

### The kynurenine pathway of tryptophan metabolism is altered in aHSCT patients compared to non-transplanted controls, resulting in accumulation of neurotoxic metabolites

**Fig. 5:**
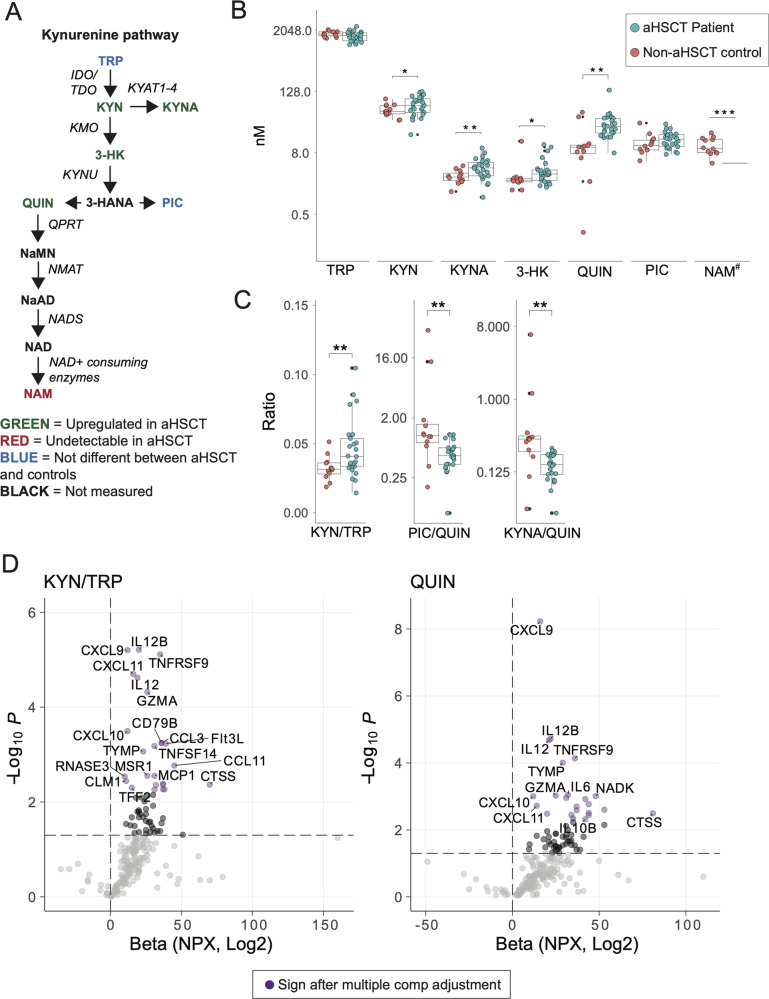
The kynurenine pathway in the CSF of all aHSCT recipients compared to non-transplanted controls. **A** Overview of the kynurenine pathway. **B** Levels of KP metabolites in aHSCT patients compared to NINDC. **C** A higher KYN/TRP ratio suggested increased activity in the rate-limiting enzymes IDO or TDO. QUIN is considered neurotoxic. PIC and KYNA are considered neuroprotective. **D** KYN/TRP and QUIN levels correlated to several proteins normally secreted by monocytes, macrophages and fibroblasts (Supplemental table 8). Purple dots indicate significant correlations after Benjamini–Hochberg correction for multiple comparisons.

Tryptophan is a precursor to the neurotransmitter serotonin, the hormone melatonin, and vitamin B3. This synthesis is distinct from the kynurenine pathway of tryptophan metabolism (Fig. 5A), a potent negative regulator of both innate and adaptive inflammation inducing long-term immune tolerance [43]. However, an imbalance in the downstream metabolism of kynurenine can result in a shift away from neuroprotective KYNA and PIC, resulting in accumulation of neurotoxic QUIN and 3-HK, and an increase in oxidative stress [44, 45].

Protein levels of kynurenine aminotransferase-1, an enzyme that synthesizes neuroprotective KYNA was downregulated in the CSF of patients with cognitive dysfunction (Fig. 3A). To further investigate possible post-transplant neurotoxicity, we assessed metabolites of the kynurenine pathway in CSF and plasma from aHSCT patients as well as from age-matched, non-transplanted, NINDC subjects. Compared to the controls, all aHSCT patients had higher concentrations of kynurenine, 3-HK and QUIN (Fig. 5A, B), suggesting increased kynurenine pathway activity. The findings were similar in all subgroups analysed, irrespective of fatigue and cognitive symptoms (Supplemental Fig. 4).

We next compared the balance between neuroprotective and toxic tryptophan metabolites. The increased kynurenine/tryptophan ratio in aHSCT patients indicated activation of the rate-limiting enzymes indoleamine deoxygenase (IDO) and/or tryptophan 2,3-dioxygenase (TDO, Fig. 5C). Importantly, in aHSCT patients, products of IDO and TDO were preferentially metabolised to QUIN rather than to KYNA and PIC, as seen in controls. This shift was specific for CSF and not observed in plasma (Fig. 5C, Supplemental Fig. 5).

Metabolism of QUIN to NAD+ has profound effects on macrophage immune responses and resolution of inflammation [46]. Notably, although the level of QUIN was elevated, the level of NAM, the breakdown product of NAD+, was undetectable in all aHSCT patients, but were readily detected in 11/12 non-transplanted controls (Fig. 5B). Multiple comparison correction revealed that, in aHSCT patients, the kynurenine/tryptophan ratio and amount of QUIN correlated to levels of monocyte, macrophage and fibroblast-derived proteins (Fig. 5D and Supplemental table 8), suggesting an association between activation of these cells in the CSF and increased KP activity [47].

### T and NK cell activation is inversely correlated to immune regulation and neuroregenerative capacity in the CNS

**Fig. 6:**
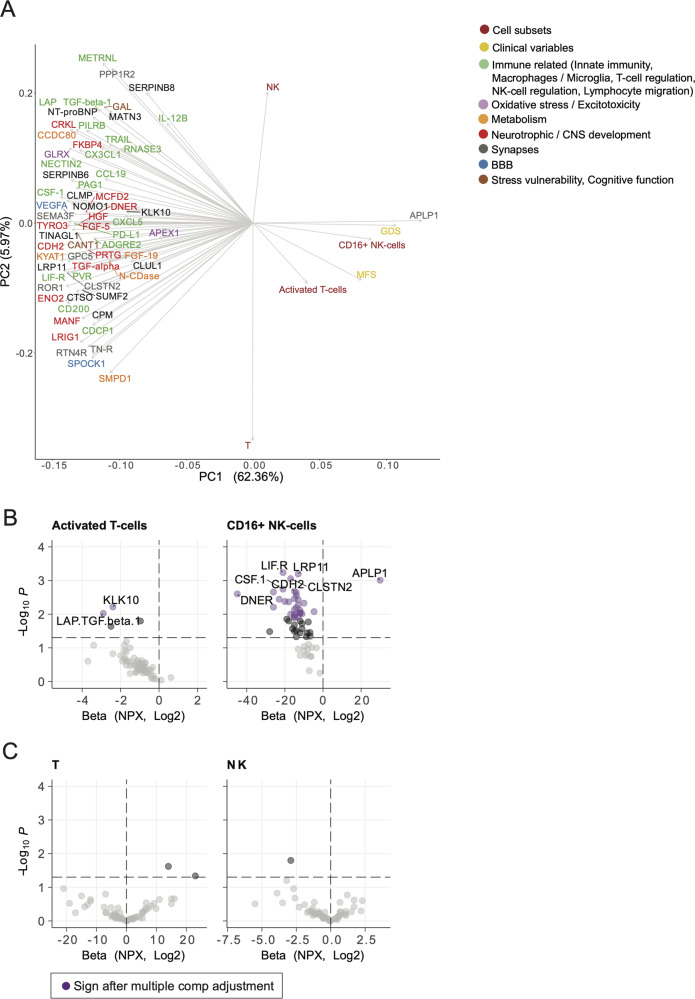
PCA and correlations between protein expression, activated T-cells and CD16^+^ NK-cells. **A** A PCA loading plot including differentially expressed proteins, immune cell subsets and clinical variables. Factors with loadings that point in the same direction suggest positive correlation and opposing factors suggest a negative correlation. **B**, **C** Volcano plots of correlations between immune cell subsets and protein expression, based on linear regression analysis. As a control, the same correlations were performed with total NK-cells and non-activated T-cells. Purple dots indicate significant correlations after Benjamini-Hochberg correction for multiple comparisons. None of the significant correlations with activated T-cells and CD16 + NK-cells were present in the control cell populations. TGF Transforming Growth Factor, PCA Principal Component Analysis, GDS Global Deficit Scale, MFS Mental Fatigue Scale, BBB Blood-Brain Barrier.

We next visualized the relationship between CSF protein concentration, immune cell subsets and clinical variables using principal component analysis (Fig. 6A). Activated T- and NK cells clearly separated from down-regulated proteins in the PC space, where clinical data and immune cell activation overlaps. These data suggested an imbalance between increased activation of innate and adaptive immune cells and reduced neuro- and immune-protective proteins. Correlation analyses between CSF proteins and activated T- or CD16+NK-cells were subsequently performed (Fig. 6B). As a control, similar correlations were performed for non-activated T-cells and total NK-cells (Fig. 6B). After multiple comparison adjustment, the activated T-cells were negatively correlated to CSF levels of TGF-β and KLK10, while the CD16^+^ NK-cells were positively correlated to APLP1 and negatively correlated to 32 proteins, including NECTIN2, LIF-R, CSF-1, CDH2 and CLSTN. Neither non-activated T-cells nor total NK-cell levels were significantly correlated to protein concentrations.

## Discussion

Cognitive impairment and fatigue are well-recognized complications of aHSCT but whether alloreactivity in the CNS plays a role remains largely unknown [48]. Our investigation of proteins, nucleic acid expression and immune cell phenotype supports persistent activation of T- and NK cells in the CSF of patients with cognitive impairment, several years after transplant, associated with a decrease in immune-regulatory proteins and increased oxidative stress. The immune activation correlated with decreased neurogenesis and impaired synaptic plasticity. These findings are in line with prefrontal cortex dysfunction observed in our previous imaging studies performed on the same patients [6].

Inflammation in the CNS can either result from activation of resident immune cells such as microglia, or from peripheral immune cells invading through different routes. Recent murine studies demonstrate that aHSCT can lead to replacement of homeostatic microglia with pro-inflammatory macrophages as well as an influx of alloreactive T-cells, causing CNS inflammation and cognitive defects [8–10].

Our clinical data are in line with these experimental models. Fatigued patients had increased levels of activated T-cells in the CSF. The subgroup of fatigued patients that suffered from cognitive impairment had additional increased levels of NK-cells expressing the activating receptor CD16. NK-cell mediated cytotoxicity has recently been demonstrated to eliminate neural stem cells in the aged hippocampus, impairing cognitive function [49]. Interestingly, mRNA sequencing revealed increased apoptotic activity in patients with cognitive dysfunction.

The increased proportion of NK-cells expressing the activating receptor CD16 were accompanied by decreased levels of nectin-2 and PVR, suggesting an imbalanced NK-cell regulation [38]. In fact, we found decreased levels of several stromal-derived, GvHD-suppressive and trophic factors in patients with cognitive dysfunction, such as TGFβ (negatively correlated to increased activated T-cell levels), HGF, CSF1, VEGF and TGFα [50, 51]. Microglial regulation was also decreased, with lower levels of the neuronally expressed CD200 and CX3CL1.

In summary, proteomics, mRNA expression and flow cytometry supported increased T-cell and NK-cell activity in patients with cognitive dysfunction, caused by lack of regulatory signalling.

T-cell activation is integral to the pathogenesis of cGvHD, and elevated effector memory T-cells are observed in the periphery at cGvHD onset [52, 53]. Further, CXCR3, which was upregulated on the activated T-cells in our study (Fig. 1), is an important chemokine receptor for homing of effector T-cells to peripheral tissues, both during cGvHD and CNS inflammation [54, 55]. However, we found no association between clinical symptoms of cGvHD and fatigue or reduced cognition, suggesting that the T-cell activation we observed was CNS-specific, rather than part of systemic cGvHD.

Neurogenesis persists in the adult hippocampus and lateral ventricles, and failure of this process is associated with risk of psychiatric disease [56, 57]. Inflammation, with activation of microglia and astrocytes, is detrimental to this process, and plays a role in the pathogenesis of neurodegeneration [58, 59]. This study demonstrates that ongoing CNS immune activity in aHSCT recipients correlated to deficient neurotrophic signalling and clinical symptoms in the form of cognitive impairments. Specifically, patients with cognitive dysfunction had decreased neurotrophic proteins such as TGFα, TGFβ, HGF, CDH2, as well as proteins calsyntenin-2 and ROR1, which have important roles in synapse formation [26–31]. The response to CNS injury was also impaired, as demonstrated by the lower levels of neuron-specific enolase, a neurotrophic protein that is normally upregulated after brain trauma [60]. A failure in neuroprotection and neurogenesis in patients with cognitive dysfunction was further corroborated by mRNA expression profiling that detected reduced levels of genes involved in cell-cell adhesion. Mechanistically, decreased AP endonuclease-1 and glutaredoxin, as well as lower expression of oxidative stress response genes (Figs. 3 and 4), suggests that the activated immune cells may impair neurogenesis in patients with cognitive dysfunction by increasing oxidative stress [59].

Proinflammatory cytokines, both in the periphery and CNS, initiate tryptophan metabolism along the kynurenine pathway by activating the rate-limiting enzymes IDO and TDO [45].

Several intermediate kynurenine metabolites are either neurotoxic (QUIN, 3-HK) or neuroprotective (KYNA, PIC), and therefore of relevance in psychiatric disorders [44, 45, 61]. Tryptophan and kynurenine readily pass through the BBB and can be further metabolised by microglia and astrocytes. In contrast, QUIN, KYNA and PIC do not pass the BBB and must be synthesized in the brain [45].

By comparing to non-transplanted controls, our study revealed increased activity of the kynurenine pathway in all aHSCT patients, both in the periphery and the CNS, without direct association to cognitive impairments or fatigue. Since the resulting increased levels of QUIN in the CSF were not counterbalanced by PIC or KYNA (demonstrated by the decreased PIC/QUIN and KYNA/QUIN ratios), the activation likely contributed to a neurotoxic environment. In plasma, the PIC/QUIN and KYNA/QUIN ratios were not different between transplanted patients and controls (Supplemental Fig. 5), suggesting that QUIN is produced intrathecally. Preclinical studies suggest that CNS macrophage activation is common after aHSCT, as donor-derived macrophages cross the BBB and adapt a microglia-like phenotype, but with increased inflammatory skewing [9, 10]. In our data, CXCL9 and other macrophage-, monocyte- and fibroblast-derived proteins in the CSF correlated to QUIN levels (Fig. 5D), consistent with previous studies suggesting a monocytic lineage origin of this metabolite [62]. Further, the end product NAM was undetectable in patient CSF, suggesting that the pathway is inhibited downstream from QUIN (Fig. 5A), as recently demonstrated in LPS-stimulated macrophages [46].

At onset of both acute and chronic GvHD, peripheral kynurenine pathway activity is stimulated by CXCL9, and correlates with disease severity [63–65]. Our results expand on these findings, by suggesting that some degree of monocytic cell activation occurs in the brain of all transplant recipients, resulting in accumulation of neurotoxic kynurenine pathway metabolites. Our proteomic data further suggests that cognitive dysfunction may result from a failure to mitigate this neurotoxicity due to lack of neurotrophic and immune-regulatory proteins.

Neurobiological aberrations underlying the previously demonstrated reduced prefrontal cortex activity in these patients [6], may ultimately lead to a persistent and relevant clinical phenotype of fatigue and cognitive dysfunction. Pathophysiological understanding of CNS disorders is limited by the invasiveness of brain biopsies. CSF sampling is a practical alternative to enable mechanistic studies through proteomic, RNA sequencing, mass spectrometric and flow cytometric analyses [66, 67]. By combining these approaches, this study demonstrates multiple lines of evidence for a failure in the regulation of CNS immune surveillance, neuroprotection, and metabolism in patients with cognitive dysfunction after aHSCT. Accordingly, exposure to oxidative stress, neurotoxic kynurenine pathway metabolites and increased NK- and T-cell activity were detected, which can all disrupt neuronal circuits responsible for higher cognitive abilities.

Larger longitudinal studies are needed to prove the cause-and-effect of an unbalanced immune activation on neurocognitive sequelae after aHSCT. Such mechanistic understanding will pave the way for targeted treatments and prevention strategies, directed at enhancing neuroprotection, synaptic plasticity, and immune regulation. Limiting the entry of kynurenine pathway metabolites to the CNS after aHSCT could also prove efficient in this regard. Importantly, this study demonstrates that CSF analyses can quantify immune dysregulation and neuronal homeostasis in the brain of aHSCT recipients. Therefore, future clinical trials of treatments for cognitive complications after aHSCT can be readily monitored using similar analyses.

## Supplementary information

Supplemental Material

## Data Availability

For original data, please contact erik.boberg@ki.se.
