# Supplementary material for: Cognitive impairments correlate with increased central nervous system immune activation after allogeneic haematopoietic stem cell transplantation
Source: Leukemia. 2023 Feb 15;37(4):888–900. doi: 10.1038/s41375-023-01840-0 (PMC10079537; doi:10.1038/s41375-023-01840-0)
Supplement: Supplementary file 1 — Supplemental Material [file 41375_2023_1840_MOESM1_ESM.docx]

**Supplemental methods**

Plasma protein profiling

Proteins were measured using the Olink® Target 96 Metabolism, Neurology and Inflammation panels (Olink Proteomics AB, Uppsala, Sweden), using a Proximity Extension Assay (PEA) technology^1^. In brief, pairs of oligonucleotide-labeled antibody probes bind to their targeted protein, and if the two probes are brought in close proximity the oligonucleotides will hybridize in a pair-wise manner. The addition of a DNA polymerase leads to a proximity-dependent DNA polymerization event, generating a unique PCR target sequence. The resulting DNA sequence is subsequently detected and quantified using a microfluidic real-time PCR instrument (Biomark HD, Fluidigm). Data is then quality controlled and normalized using an internal extension control and an inter-plate control, to adjust for intra- and inter-run variation. The final assay read-out is presented in Normalized Protein eXpression (NPX) values, which is an arbitrary unit on a log2-scale where a high value corresponds to a higher protein expression. All assay validation data (detection limits, intra- and inter-assay precision data, etc.) are available on manufacturer's website ([www.olink.com](http://www.olink.com)).

mRNA sequencing

CSF supernatant was thawed and centrifuged at 2000xg for 10 minutes. RNA was extracted as in Bost et al 202126. Briefly, 100uL of centrifuged CSF was resuspended in 400μL of TRI Reagent (Sigma-Aldrich, USA) and 100μL of chloroform was added after five minutes. The tubes were shaken vigorously for 1min. After 15min of incubation at room temperature, the samples were centrifuged for 15min at 12 000xg at 4°C. Subsequently, precipitation was performed as in Hagey et al 202127. 400μL of the aqueous phase was mixed by inversion with 400μL of isopropanol, 30μL of 3m sodium acetate, and 1μL of Pellet Paint (Merck, USA) and incubated over night at −20°C. The samples were then centrifuged for 30min at 20 000xg at 4°C and pellets were washed twice with 700μL of 70% ethanol. The pellets were then air dried and resuspended in 15μL of elution buffer (Qiagen, USA). cDNA was produced using the Smart-seq2 RNA-sequencing protocol^2^ and 50 bp single end reads were sequenced on an Illumina HiSeq 3000 (Illumina, USA). Reads were then mapped to the ENSEMBL human transcriptome GRCh37 using Tophat 2.1.1.

Ultra-performance liquid chromatography (UPLC-MS/MS)

Tryptophan (TRP), kynurenine (KYN), kynurenic acid (KYNA), quinolinic acid (QUIN), picolinic acid (PIC), 3-hydroxykynurenic acid (3-HK), nicotinamide (NAM) and nicotinic acid were quantified in CSF and plasma by UPLC-MS/MS system using a Xevo TQ-XS triple-quadrupole mass spectrometer (Waters, Manchester, UK) equipped with a Z-spray electrospray interface and a Waters Acquity UPLC I-Class FTN system (Waters, MA, USA). Full description of the CSF and plasma UPLC-MS/MS method, sample preparation and stability test of all metabolites can be found in Schwieler et al., 2020^3^ and Trepci et al., 2020^4^. In brief, the MS was operated in electrospray-positive multiple reaction monitoring (MRM) mode with a source temperature of 150 °C, capillary voltage of + 3.0 kV, desolvation temperature 650 °C, desolvation gas flow rate 1000 l/h and detector gain 1. Used column was Acquity HSS T3 2.1 × 150 mm, 1.8 µm (Waters, Product Number [PN]: 186,003,540) in a temperature of 50 °C. The two mobile phases were composed of A: 0.6% formic acid in water and B: 0.6% formic acid in methanol (UPLC grade). An isolator column (Waters, 2.1 × 50 mm column, PN: 186,004,476) was installed to retain contaminants from the mobile phase. The flow rate was set at 0.3 ml/min and the run time for each sample was 13.0 min. The m/z for the MRM transitions of each individual analyte were: KYN, 209 > 94; KYNA, 190 > 116; QUIN, 168 > 78; PIC, 124 > 78; NAM, 123 > 78; 3-HK, 225 > 110; Nicotinic acid, 124 > 80 and for the internal standards (IS): KYN-d4, 213 > 94; QUIN-d3, 171 > 81; KYNA-d5, 195 > 121; PIC-d4, 128 > 82; NAM-[13C6], 129 > 101; 3-HK- d3, 228 > 163 and Nicotinic acid-[13C6], 130 > 85. Nicotinic acid was detected in less than 50% of all samples and was included in the method to make sure we could distinguish it from the isomer PIC.

All metabolites measured in CSF and plasma samples were detected in higher concentrations than lowest level of detection in plasma (LLOD, Tryptophan, 50 nM kynurenine, 50 nM; KYNA, 5 nM; QUIN, 5 nM; PIC, 5 nM; 3-HK, 5 nM; NAM, 5 nM) and CSF (LLOD, Tryptophan, 1 nM kynurenine, 1 nM; KYNA, 1 nM; QUIN, 1 nM; PIC, 5 nM; 3-HK, 1 nM; NAM, 5 nM). The inter-assay variation (%CV) between samples analysed in duplicates within a day (intra-assay) were less than 5% for all metabolites measured. The variation between two different experiments running over 2 days (inter-assay, n=2) were less than 6% for all metabolites.

**Statistical analysis**

Homogeneity of variance

Equality of variance between the study groups was assessed using Levene’s test. Compared groups had homogeneous variance in 96% of the proteomic comparisons, 98% of the cell subset comparisons and 92% of the kynurenine pathway metabolite comparisons. The deseq2 package used for analysing mRNA expression data handles inter-group variation internally.

**Supplemental table 1: Inclusion and exclusion criteria**

| **#** | **Inclusion criteria** | **Exclusion criteria** |
| --- | --- | --- |
| 1 | ≥ 18 years old | History of intracranial infection |
| 2 | Underwent allogeneic HSCT for hematologic malignancy ≥12months and ≤5 years ago. | History of intrathecal chemotherapy |
| 3 |  | Treated with total body irradiation as part of conditioning regiment for HSCT. |
| 4 |  | Current active or chronic neurological or psychiatric disorder, such as stroke, inflammatory neurological disease, schizophrenia, severe depression, suicide tendencies, anorexia nervosa, severe mood swings, bipolar disorder or any type of dementia or other degenerative neurological disease. |
| 5 |  | Current use of antipsychotic drugs, tricyclic antidepressants (in higher doses used for treating depression), high doses of benzodiazepines (20mg of diazepam daily or equivalent), high doses of opioids (30mg of morphine or equivalent daily). |
| 6 |  | Current or history of substance abuse. |
| 7 |  | Current medication with steroids, ≥15mg of prednisolone or equivalent. |
| 8 |  | MRI showing signs of elevated intracranial pressure or increased risk of cerebral herniation following lumbar puncture. |
| 9 |  | Increased risk of bleeding, i.e. thrombocytes < 50 x 10^9^/L, Prothrombin Time, International Normalized Ratio > 1.4, complicating lumbar puncture. |
| 10 |  | Skin infection at the location for lumbar puncture (above the L3/L4 or L4/L5 lumbar vertebra). |
| 11 |  | Magnetic implant, or implanted device disturbing MRI examination. |

**Supplemental table 2: Baseline characteristics of the NINDC and the aHSCT recipients**

| **Variable** | **aHSCT recipients (n=25)** | **NINDC (n=12)** | **p** |
| --- | --- | --- | --- |
| **Age (years):** median (range) | 57 (22-73) | 52.5 (47-60) | 0.89^a^ |
| **Sex (M):** num (%) | 11 (44) | 5 (42) | 1^b^ |
| **BMI (kg/m^2^):** mean (sd) | 24.8 (2.5) | 25.6 (3.2) | 0.46^c^ |

^a^Wilcoxon rank-sum test, ^b^Fisher’s exact test, ^c^T-test. NINDC: Non-inflammatory Neurological Disease Controls

**Supplemental table 3: Included proteomic assays**

| **Inflammation** | | **Metabolism** | | **Neurology** | |
| --- | --- | --- | --- | --- | --- |
| **Protein** | **% below LOD^#^** | **Protein** | **% below LOD ^#^** | **Protein** | **% below LOD ^#^** |
| 4E-BP1 | 0 | ACP6 | 0 | ADAM 22 | 0 |
| ADA | 0 | ADGRE2 | 0 | ADAM 23 | 0 |
| CCL11 | 0 | ADGRG2 | 0 | Alpha-2-MRAP | 0 |
| CCL19 | 0 | ANGPT2 | 0 | BCAN | 0 |
| CCL23 | 0 | ANGPTL1 | 0 | BMP-4 | 0 |
| CCL3 | 0 | ANGPTL7 | 0 | CADM3 | 0 |
| CCL4 | 0 | APEX1 | 0 | CD200 | 0 |
| CD40 | 0 | APLP1 | 0 | CD200R1 | 0 |
| CD5 | 0 | BAG6 | 0 | CD38 | 0 |
| CD8A | 0 | CANT1 | 0 | CDH3 | 0 |
| CDCP1 | 0 | CCDC80 | 0 | CDH6 | 0 |
| CSF-1 | 0 | CD164 | 0 | CLM-1 | 0 |
| CST5 | 0 | CD1C | 0 | CLM-6 | 0 |
| CXCL1 | 0 | CD2AP | 0 | CNTN5 | 0 |
| CXCL10 | 0 | CD79B | 0 | CPA2 | 0 |
| CXCL11 | 0 | CDH2 | 0 | CPM | 0 |
| CXCL5 | 0 | CDHR5 | 0 | CTSC | 0 |
| CXCL6 | 0 | CHRDL2 | 0 | CTSS | 0 |
| CXCL9 | 0 | CLEC5A | 0 | DDR1 | 0 |
| DNER | 0 | CLMP | 0 | Dkk-4 | 0 |
| FGF-19 | 0 | CLSTN2 | 0 | DRAXIN | 0 |
| Flt3L | 0 | CLUL1 | 0 | EDA2R | 0 |
| HGF | 0 | CRKL | 0 | EFNA4 | 0 |
| IL-10RB | 0 | CTSH | 0 | EPHB6 | 0 |
| IL-12B | 0 | CTSO | 0 | EZR | 0 |
| IL-18R1 | 0 | DDC | 0 | FLRT2 | 0 |
| IL18 | 0 | DPP7 | 0 | gal-8 | 0 |
| IL6 | 0 | ENO2 | 0 | GCP5 | 0 |
| IL7 | 0 | ENPP7 | 0 | GDF-8 | 0 |
| IL8 | 0 | ENTPD5 | 0 | GDNFR-alpha-3 | 0 |
| LAP TGF-beta-1 | 0 | FAM3C | 0 | GFR-alpha-1 | 0 |
| LIF-R | 0 | FCRL1 | 0 | GM-CSF-R-alpha | 0 |
| MCP-1 | 0 | GAL | 0 | GZMA | 0 |
| MCP-2 | 0 | GLRX | 0 | IL12 | 0 |
| MCP-4 | 0 | IGFBPL1 | 0 | JAM-B | 0 |
| MMP-1 | 0 | KLK10 | 0 | KYNU | 0 |
| MMP-10 | 0 | KYAT1 | 0 | LAIR-2 | 0 |
| OPG | 0 | LRIG1 | 0 | LAYN | 0 |
| PD-L1 | 0 | LRP11 | 0 | MANF | 0 |
| SCF | 0 | MCFD2 | 0 | MATN3 | 0 |
| TGF-alpha | 0 | METRNL | 0 | MDGA1 | 0 |
| TNFB | 0 | NADK | 0 | MSR1 | 0 |
| TNFRSF9 | 0 | NECTIN2 | 0 | N2DL-2 | 0 |
| TRAIL | 0 | NOMO1 | 0 | NAAA | 0 |
| TWEAK | 0 | NPDC1 | 0 | NBL1 | 0 |
| uPA | 0 | NPTXR | 0 | NCAN | 0 |
| VEGFA | 0 | NT-proBNP | 0 | NEP | 0 |
| FGF-5 | 4 | PAG1 | 0 | Nr-CAM | 0 |
| TNFSF14 | 7 | PILRB | 0 | NRP2 | 0 |
| CCL28 | 11 | PPP1R2 | 0 | NTRK2 | 0 |
| TNF | 21 | QDPR | 0 | NTRK3 | 0 |
| CCL25 | 25 | REG4 | 0 | PDGF-R-alpha | 0 |
| CX3CL1 | 25 | RNASE3 | 0 | PLXNB1 | 0 |
| CD244 | 29 | ROR1 | 0 | PLXNB3 | 0 |
| SIRT2 | 32 | RTN4R | 0 | PRTG | 0 |
| **CCL20** | **50** | SDC4 | 0 | PVR | 0 |
| **CD6** | **50** | SEMA3F | 0 | RGMA | 0 |
| **LIF** | **50** | SERPINB6 | 0 | RGMB | 0 |
| **EN-RAGE** | **64** | SERPINB8 | 0 | ROBO2 | 0 |
| **IFN-gamma** | **64** | SIGLEC7 | 0 | SCARA5 | 0 |
| **OSM** | **64** | SOST | 0 | SCARB2 | 0 |
| **STAMBP** | **64** | SUMF2 | 0 | SCARF2 | 0 |
| **AXIN1** | **86** | TFF2 | 0 | sFRP-3 | 0 |
| **FGF-21** | **86** | THOP1 | 0 | Siglec-9 | 0 |
| **ARTN** | **89** | TINAGL1 | 0 | SIGLEC1 | 0 |
| **IL-20RA** | **89** | TXNDC5 | 0 | SKR3 | 0 |
| **IL10** | **89** | TYMP | 0 | SMOC2 | 0 |
| **IL-1 alpha** | **93** | TYRO3 | 0 | SMPD1 | 0 |
| **IL-15RA** | **93** | VCAN | 0 | SPOCK1 | 0 |
| **IL-17A** | **93** | FKBP4 | 7 | THY 1 | 0 |
| **IL-17C** | **93** | TSHB | 11 | TMPRSS5 | 0 |
| **IL5** | **93** | ANXA11 | 14 | TN-R | 0 |
| **NRTN** | **93** | GHRL | 14 | TNFRSF12A | 0 |
| **NT-3** | **93** | LILRA5 | 18 | TNFRSF21 | 0 |
| **SLAMF1** | **93** | SSC4D | 18 | UNC5C | 0 |
| **TRANCE** | **93** | USP8 | 21 | VWC2 | 0 |
| **TSLP** | **93** | DIABLO | 25 | CLEC1B | 4 |
| **CASP-8** | **96** | NQO2 | 29 | N-CDase | 4 |
| **FGF-23** | **96** | SNAP23 | 29 | MAPT | 7 |
| **GDNF** | **96** | MEP1B | 32 | RSPO1 | 7 |
| **IL-10RA** | **96** | S100P | 36 | WFIKKN1 | 11 |
| **IL13** | **96** | **ALDH1A1** | **54** | CRTAM | 18 |
| **IL4** | **96** | **ITGB7** | **54** | NMNAT1 | 32 |
| **Beta-NGF** | **100** | **CA13** | **57** | **CLEC10A** | **68** |
| **IL-20** | **100** | **HDGF** | **57** | **GDNF** | **75** |
| **IL-22 RA1** | **100** | **GRAP2** | **64** | **IL-5R-alpha** | **82** |
| **IL-24** | **100** | **ANXA4** | **79** | **Beta-NGF** | **89** |
| **IL-2RB** | **100** | **DAB2** | **79** | **FcRL2** | **89** |
| **IL2** | **100** | **AHCY** | **82** | **G-CSF** | **93** |
| **IL33** | **100** | **COMT** | **89** | **HAGH** | **93** |
| **MCP-3** | **100** | **FBP1** | **93** | **LAT** | **93** |
| **ST1A1** | **100** | **ARG1** | **100** | **LXN** | **93** |

# The % of samples with levels below the limit of detection (LOD). Proteins in bold were below the LOD in at least 50% of the samples and were consequently excluded from further analysis.

**Supplemental table 4:** **Composition of the flow cytometry panel**

| **Laser** | **Fluorochrome** | **Marker** | **Catalogue number^#^** | **Concentration** |
| --- | --- | --- | --- | --- |
| **Ultra-**  **violet** | **BUV805** | **HLA-DR** | 752497 | 1/100 |
|  | **BUV737** | **PD-1 (CD279)** | 612791 | 1/100 |
|  | **BUV661** | **CD6** | 750775 | 1/100 |
|  | **BUV615** | **CD56** | 751387 | 1/50 |
|  | **BUV563** | **CCR7** | 741317 | * |
|  | **BUV496** | **CD14** | 750381 | 1/100 |
|  | **BUV395** | **CD206** | 740309 | 1/100 |
| **Violet** | **BV786** | **CTLA-4 (CD152)** | 563931 | 1/100 |
|  | **BV750** | **CD86** | 747383 | 1/100 |
|  | **BV711** | **CD16** | 563127 | 1/100 |
|  | **BV650** | **ST2** | 745403 | 1/100 |
|  | **BV605** | **CD163** | 745091 | 1/100 |
|  | **BV570** | **CD4** | 300534 | 1/100 |
|  | **V500** | **CELL DEATH MARKER** | L34957 | 1/100 |
|  | **V500** | **CD19** | 561125 | 1/25 |
|  | **BV421** | **CXCR6** | 566008 | 1/100 |
| **Blue** | **BB515 (FITC)** | **CD96** | 564774 | 1/100 |
|  | **BB700 (PerCP Cy5.5)** | **CD8** | 566451 | 1/200 |
| **Yellow-green** | **PE-cy7** | **CD69** | 335792 | 1/100 |
|  | **PE-cy5** | **CXCR3 (CD183)** | 335792 | 1/100 |
|  | **PE-CF495** | **CD11c** | 566836 | 1/100 |
|  | **PE** | **CD118** | 566384 | 1/100 |
| **Red** | **APC-H7** | **CD44** | 560532 | 1/100 |
|  | **Alexa700** | **CD3** | 561027 | 1/100 |
|  | **Alexa647** | **CX3CR1** | 565894 | 1/100 |

* 1ul of CCR7 was added to the cell pellet in each well and incubated at 37^o^C for 30 minutes, before adding the master mix of all other antibodies and incubating again for 20 minutes at 4^o^C

^#^ All antibodies were acquired from BD Biosciences, except for two: CD4 (BV570) was acquired from BioLegend and the CELL DEATH MARKER was acquired from Thermo Fisher.

**Supplemental table 5: Flow cytometry data comparisons, fatigue vs non-fatigue**

| **Subset** | **Shapiro Wilk’s test** | **Levene’s test** | **Sign test** | **p** |  |
| --- | --- | --- | --- | --- | --- |
| Lymphocytes | p=0.0074 | p=0.84 | a | 0.37 | ns |
| NK-cells | p=0.5 | p=0.76 | b | 0.8 | ns |
| CD56loD16hi NK | p=0.38 | p=0.44 | b | 0.73 | ns |
| CD56hiCD16lo NK | p=0.96 | p=0.48 | b | 0.56 | ns |
| CD16neg NK | p=0.88 | p=0.41 | b | 0.7 | ns |
| CD16pos NK | p=0.88 | p=0.89 | b | 0.3 | ns |
| NK: PD1 MFI | p=0.00033 | p=0.45 | a | 0.29 | ns |
| NK: CXCR6 MFI | p=0.72 | p=0.43 | b | 0.36 | ns |
| NK: CD96 MFI | p=0.97 | p=0.24 | b | 0.92 | ns |
| NK: CXCR3 MFI | p=0.25 | p=0.61 | b | 0.96 | ns |
| NK: CD69 MFI | p=0.43 | p=0.25 | b | 0.31 | ns |
| NKT | p=0.00029 | p=0.57 | a | 0.96 | ns |
| T % of lymphocytes | p=0.008 | p=0.82 | a | 0.17 | ns |
| T: CX3CR1 MFI | p=0.81 | p=0.35 | b | 0.36 | ns |
| T: CD44 MFI | p=0.29 | p=0.22 | b | 0.83 | ns |
| T: CCR7 MFI | p=0.53 | p=0.11 | b | 0.21 | ns |
| T: CD6 MFI | p=0.96 | p=0.7 | b | 0.76 | ns |
| T: PD1 MFI | p=0.53 | p=0.39 | b | 0.45 | ns |
| T: HLA-DR MFI | p=0.11 | p=0.89 | b | 0.69 | ns |
| T: CXCR6 MFI | p=0.0034 | p=0.83 | a | 0.87 | ns |
| T: ST2 MFI | p=0.67 | p=0.26 | b | 0.9 | ns |
| T: CD118 MFI | p=0.71 | p=0.83 | b | 0.67 | ns |
| T: CXCR3 MFI | p=0.61 | p=0.14 | b | 0.3 | ns |
| T: CD69 MFI | p=0.37 | p=0.47 | b | 0.74 | ns |
| CD4+ T | p=0.71 | p=0.97 | b | 0.98 | ns |
| CD8+CD8+ T | p=0.89 | p=0.62 | b | 0.56 | ns |
| CD8+ T | p=0.83 | p=0.99 | b | 0.75 | ns |
| CD4-CD8- T | p=0.17 | p=0.56 | b | 0.39 | ns |
| CD3-CD56- lymphocytes | p=2.3e-05 | p=0.73 | a | 0.028 | * |
| Activated T % of live | p=0.88 | p=0.53 | b | 0.016 | * |
| Activated T: CX3CR1 MFI | p=0.56 | p=0.75 | b | 0.11 | ns |
| Activated T: CD44 MFI | p=0.0038 | p=0.94 | a | 0.11 | ns |
| Activated T: CCR7 MFI | p=0.0039 | p=0.58 | a | 0.51 | ns |
| Activated T: CD6 MFI | p=0.85 | p=0.21 | b | 0.44 | ns |
| Activated T: PD1 MFI | p=1 | p=0.36 | b | 0.71 | ns |
| Activated T: HLA-DR MFI | p=0.67 | p=0.15 | b | 0.22 | ns |
| Activated T: CXCR6 MFI | p=0.14 | p=0.049 | b | 0.99 | ns |
| Activated T: ST2 MFI | p=0.43 | p=0.94 | b | 0.32 | ns |
| Activated T: CD118 MFI | p=0.023 | p=0.82 | a | 0.71 | ns |
| Activated T: CXCR3 MFI | p=0.25 | p=0.46 | b | 0.97 | ns |
| Activated T: CD69 MFI | p=2.9e-05 | p=1 | a | 0.76 | ns |
| CD4+ Activated T | p=0.78 | p=0.69 | b | 0.69 | ns |
| CD4+CD8+ Activated T | p=0.48 | p=0.34 | b | 0.0033 | ** |
| CD8+ Activated T | p=0.21 | p=0.63 | b | 0.91 | ns |
| T % of live | p=0.029 | p=0.97 | a | 0.29 | ns |
| Monocytes % of live | p=0.49 | p=0.21 | b | 0.27 | ns |
| Non-classical monocytes | p=0.56 | p=0.34 | b | 0.34 | ns |
| Intermediate monocytes | p=0.0048 | p=0.78 | a | 0.45 | ns |
| Classical monocytes | p=0.79 | p=0.77 | b | 0.61 | ns |
| M2 monocytes | p=0.00085 | p=0.45 | a | 0.81 | ns |
| Monocytes: CX3CR1 MFI | p=0.18 | p=0.27 | b | 0.74 | ns |
| Monocytes: CD44 MFI | p=0.88 | p=0.86 | b | 0.31 | ns |
| Monocytes: CCR7 MFI | p=0.61 | p=0.83 | b | 0.84 | ns |
| Monocytes: HLADR MFI | p=0.27 | p=0.51 | b | 0.15 | ns |
| Monocytes: CD86 MFI | p=0.93 | p=0.29 | b | 0.48 | ns |
| mDC | p=0.15 | p=0.052 | b | 0.3 | ns |

p<0.05 on Shapiro-Wilks’ test indicates a non-normal distribution. p<0.05 on Levene’s test indicates heterogeneity of variance.
a) Wilcoxon rank sum test, b) Student’s t-test

**Supplemental table 6: Flow cytometry data comparisons, CD vs non-CD**

| **Subset** | **Shapiro Wilk's test** | **Levene's test** | **Sign test** | **p** |  |
| --- | --- | --- | --- | --- | --- |
| Lymphocytes | p=0.0013 | p=0.47 | a | 0.85 | ns |
| NK-cells | p=0.0034 | p=0.43 | a | 0.47 | ns |
| CD56loD16hi NK | p=0.0013 | p=0.9 | a | 0.35 | ns |
| CD56hiCD16lo NK | p=0.89 | p=0.57 | b | 0.5 | ns |
| CD16neg NK | p=4.2e-06 | p=0.65 | a | 0.045 | * |
| CD16pos NK | p=0.012 | p=0.88 | a | 0.017 | * |
| NK: PD1 MFI | p=0.61 | p=0.2 | b | 0.29 | ns |
| NK: CXCR6 MFI | p=0.075 | p=0.64 | b | 0.52 | ns |
| NK: CD96 MFI | p=0.3 | p=0.69 | b | 0.34 | ns |
| NK: CXCR3 MFI | p=0.036 | p=1 | a | 0.26 | ns |
| NK: CD69 MFI | p=0.41 | p=0.58 | b | 0.73 | ns |
| NKT | p=0.023 | p=0.72 | a | 0.63 | ns |
| T % of lymphocytes | p=0.0022 | p=0.53 | a | 0.68 | ns |
| T: CX3CR1 MFI | p=0.53 | p=0.23 | b | 0.018 | * |
| T: CD44 MFI | p=0.032 | p=0.69 | a | 0.61 | ns |
| T: CCR7 MFI | p=0.044 | p=0.22 | a | 0.44 | ns |
| T: CD6 MFI | p=0.69 | p=0.96 | b | 0.29 | ns |
| T: PD1 MFI | p=0.59 | p=0.47 | b | 0.64 | ns |
| T: HLA-DR MFI | p=0.26 | p=0.71 | b | 0.63 | ns |
| T: CXCR6 MFI | p=0.025 | p=0.42 | a | 0.55 | ns |
| T: ST2 MFI | p=1 | p=0.73 | b | 0.21 | ns |
| T: CD118 MFI | p=0.081 | p=0.58 | b | 0.85 | ns |
| T: CXCR3 MFI | p=6.2e-06 | p=0.25 | a | 0.44 | ns |
| T: CD69 MFI | p=0.24 | p=0.6 | b | 0.14 | ns |
| CD4+ T | p=0.47 | p=0.39 | b | 0.49 | ns |
| CD8+CD8+ T | p=0.64 | p=0.076 | b | 0.084 | ns |
| CD8+ T | p=0.53 | p=0.34 | b | 0.42 | ns |
| CD4-CD8- T | p=1.8e-06 | p=0.94 | a | 0.29 | ns |
| CD3-CD56- lymphocytes | p=3.3e-06 | p=0.35 | a | 0.06 | ns |
| Activated T % of live | p=0.061 | p=0.16 | b | 0.0086 | ** |
| Activated T: CX3CR1 MFI | p=0.97 | p=0.45 | b | 0.74 | ns |
| Activated T: CD44 MFI | p=0.13 | p=0.64 | b | 0.66 | ns |
| Activated T: CCR7 MFI | p=0.0017 | p=0.86 | a | 0.67 | ns |
| Activated T: CD6 MFI | p=0.25 | p=0.66 | b | 0.52 | ns |
| Activated T: PD1 MFI | p=0.55 | p=0.62 | b | 0.39 | ns |
| Activated T: HLA-DR MFI | p=0.0014 | p=0.29 | a | 0.54 | ns |
| Activated T: CXCR6 MFI | p=0.066 | p=0.22 | b | 0.6 | ns |
| Activated T: ST2 MFI | p=0.9 | p=0.79 | b | 0.99 | ns |
| Activated T: CD118 MFI | p=0.037 | p=0.89 | a | 1 | ns |
| Activated T: CXCR3 MFI | p=0.0066 | p=0.38 | a | 0.72 | ns |
| Activated T: CD69 MFI | p=1.1e-05 | p=0.28 | a | 0.49 | ns |
| CD4+ Activated T | p=0.41 | p=0.7 | b | 0.65 | ns |
| CD4+CD8+ Activated T | p=0.018 | p=0.31 | a | 0.32 | ns |
| CD8+ Activated T | p=0.32 | p=0.73 | b | 0.87 | ns |
| T % of live | p=0.0071 | p=0.44 | a | 0.93 | ns |
| Monocytes % of live | p=0.0049 | p=0.92 | a | 0.83 | ns |
| Non-classical monocytes | p=1.5e-08 | p=0.57 | a | 0.73 | ns |
| Intermediate monocytes | p=0.52 | p=0.88 | b | 0.85 | ns |
| Classical monocytes | p=0.015 | p=0.78 | a | 0.2 | ns |
| M2 monocytes | p=0.7 | p=0.031 | b | 0.34 | ns |
| Monocytes: CX3CR1 MFI | p=0.00099 | p=0.59 | a | 0.97 | ns |
| Monocytes: CD44 MFI | p=0.05 | p=0.97 | a | 0.64 | ns |
| Monocytes: CCR7 MFI | p=0.47 | p=0.81 | b | 0.72 | ns |
| Monocytes: HLADR MFI | p=0.38 | p=0.59 | b | 0.79 | ns |
| Monocytes: CD86 MFI | p=0.52 | p=0.66 | b | 0.68 | ns |
| mDC | p=0.025 | p=0.41 | a | 0.75 | ns |

p<0.05 on Shapiro-Wilks’ test indicates a non-normal distribution. p<0.05 on Levene’s test indicates heterogeneity of variance.
a) Wilcoxon rank sum test, b) Student’s t-test

**Supplemental table 7: Categorization of differentially expressed proteins based on literature search**

| **Protein** | **Comment** |
| --- | --- |
| *Blood-brain barrier* | |
| **SPOCK1** | SPOCK1 suggested as neuronal marker that signals to BBB endothelium. Lack of SPOCK1 causes ECM malformation and increased BBB leakage / disrupted pericyte-endothelial cell communication^5^. |
| **VEGFA** | Vital for embryonic development of BBB (as other blood vessels). Can cause increased BBB permeability in MS or epilepsy^6^. Stimulates neurogenesis^7^. |
| *Cognitive function* | |
| **CANT1** | Mutation causes Desbuquois dysplasia, with growth restriction and cognitive impairment^8^. Mechanism behind cognitive impairment not clear. |
| *Innate immunity* | |
| **CXCL5** | Chemokine for neutrophils^9^. Protects from sepsis^9^. Secreted by macrophages and eosinophils, but also fibroblasts^10^. |
| **PILRB** | Regulatory for innate immune cells. Largely unknown function in host defence/inflammation. Seems to activate macrophages to produce more inflammatory cytokines, but at the same time decrease bacterial clearance^11^. |
| **RNASE3** | Secreted by eosinophils. Neurotoxic, fibrosis-promoting, immune-regulatory. Stimulates TGFb release from fibroblasts. Inhibits T-cell growth and production from B-cells/plasma cells^12^. |
| *Lymphocyte migration* | |
| **CCL19** | Binds CCR7. Attracts T-cells to secondary lymphoid organs^13^. Elevated in MS. Constituitively expressed in healthy CSF. Likely produced in the brain, possibly by monocytes. Important for immune surveillance in CNS, and for maintaining pathologic T-cells in MS^14^. |
| **CDCP1** | Attracts T-cells with the CD6 receptor^15^. Perhaps important in MS and RA. |
| *Macrophages/Microglia* | |
| **CD200** | CD200-CD200R interaction between neurons and microglia controls microglia activation. Neurons express CD200. Expression of CD200 goes down early in EAE^16^. |
| **CSF-1** | Recruits macrophages/microglia, promotes proliferation and convertion to M2 phenotype. May induce removal of debris from the brain through increased phagocytosis^17^. |
| **CX3CL1** | Expressed by neurons. Interacts with microglia through CX3CR1. Can be neuroprotective, but also sustain neuroinflammation and contribute to neurotoxicity^18^. |
| **ADGRE2** | Plays a role during myeloid cell migration, adhesion, activation. Unspecified role^19^. |
| **METRNL** | Epressed by macrophages and mucosa. Secretion inhibited by TGFb. METRNL-/- mice develops inflammatory lesions. Contributes to control of inflammation, likely via macrophage regulation^20^. Also upregulated after exercise, increases energy expenditure, linked to brown fat^21^. Similar structure to meteorin (but different protein) that is highly expressed in the CNS and involved in glial cell differentiation and negative feedback in gliosis. |
| *Metabolism* | |
| **N-CDase** | Neutral ceramidase^22^. |
| **KYAT1** | Metabolizes kynurenine to neuroprotective kynurenic acid^23^. |
| **CCDC80** | Possibly involved in glucose/energy homeostasis, diet-induced obesity^24^. |
| **FGF-19** | Acts on b-klotho on nerves in the hypothalamus to induce weight loss and lower glucose/insulin levels^25,26^. |
| **SMPD1** | Encodes acid sphingomyelinase, which breaks down sphingomyelin. Genetic deficiency causes accumulation of lipids in macrophages/monocytes and in ganglion cells in CNS leading to Niemann-Pick disease^27^. |
| *Neurotrophic / CNS development* | |
| **CDH2** | Cell-cell adhesion. Involved in neuronal migration and axon pathfinding^28^. |
| **CRKL** | Involved in the Reelin pathway that is important for brain development^29^. |
| **DNER** | Important for cerebellum development via the notch signalling pathway^30^. |
| **ENO2** | Released after neuronal injury. Promotes ECM degradation and inflammation. Also can have neurotrophic functions as it controls neuronal survival, differentiation, and neurite generation^31^. |
| **FGF-5** | Neurotrophic actions on cholinergic and raphe serotonergic neurons, in rat hippocampus^32^. |
| **FKBP4 (FKBP52)** | Controls neuronal growth cones^33^. |
| **HGF** | Increases differentiation, survival and axonal outgrowth in neurons. Also T-cell suppression^34,35^. |
| **LRIG1** | Dampens neuronal stem cell responses to EGF by inhibiting EGF-R. Decreases proliferation of neuronal stem cells and promotes their transition to adult neuronal stem cells postnatally^36^. |
| **MANF** | Promotes dopaminergic neuron survival^37^. |
| **MCFD2** | Hippocampal adult neurogenesis^38^. |
| **PRTG** | Important during early embryonic neural development^39^. |
| **TGF-alpha** | Acts on EGF-R and has trophic actions on neurons^40,41^. |
| **TYRO3** | Increases CNS myelin generation^42^. |
| *NK-cell regulation* | |
| **PVR** | CD155 - Ligand for DNAM (CD226) and CD96. Expressed by APCs. DNAM binding activates NK-cell mediated lysis. CD96 and TIGIT binding inhibits NK-cell mediated lysis^43,44^. |
| **NECTIN2** | CD112 - Ligand for DNAM (CD226). Expressed by APCs. DNAM binding activates NK-cell mediated lysis. TIGIT binding inhibits NK-cell mediated lysis^43^. |
| *Oxidative stress / Excitotoxicity* | |
| **APEX1** | Increased expression during oxidative stress. Protects neurons from oxidative stress in Zebrafish^45^. |
| **GLRX** | Downregulation increases sensitivity to excitotoxicity^46^. |
| *Stress vulnerability* | |
| **GAL** | Gene variants in GAL increases vulnerability to psychological stress^47^. |
| *Synapses* | |
| **APLP1** | Expressed both pre- and postsynaptically. Expression is important for synaptic maintenance^48^. |
| **CLSTN2** | Lack of CLSTN2 leads to disrupted synaptic density, shape and connectivity^49^. |
| **GPC5** | Glypicans important for synaptic development and neural plasticity^50^. |
| **PPP1R2** | Regulates memory formation by regulating synaptic plasticity^51^. |
| **ROR1** | Downregulation leads to decreased synapse formation in hippocampus^52^. |
| **RTN4R** | Downregulates formation of excitatory synapses in the hippocampus. RTN4R is downregulated by neuronal activity, which may lead to activity dependent synapse formation^53^. |
| **SEMA3F** | Negative regulator of synapse formation and spine development in the postnatal CNS. Loss leads to aberrant synapse formation^1–8154^. |
| **TN-R** | Brain ECM protein. Inhibition decreases synaptic vesicle exo- and endocytosis, disrupting synaptic communication^55^. |
| *T-cell regulation* | |
| **PD-L1** | Immune checkpoint molecule. Binds PD1 on T-cells, B-cells and myeloid cells. Decreases activation^56^. |
| **IL-12B** | Promotes Th1, INFg production, activates NK-cells^57^. |
| **LIF-R** | CD112R. Expressed on T-cells, binding of CD112 reduces T-cell activation^58^. Relevant in multiple sclerosis^59^. |
| **PAG1** | Transmembrane protein on T-cells. Transmits regulatory signals and helps keep the resting T-cells in a quiescent state^60^. |
| **LAP TGF-beta-1** | Regulates T-cell development in thymus, T-cell tolerance to peripheral antigens and T-cell differentiation during the immune response^61^. Enhances survival of newly formed neurons and regulates neuronal stem cell quiescence^62^. |
| **TRAIL** | Inhibits autoimmune disease by inhibiting Th1-cells and promoting Tregs^63^. Can reduce MS/EAE pathology^64^. |
| *Unknown / other* | |
| **CPM** | Peptidase. Proposed role in inflammation. Expressed in the brain^65^. |
| **CTSO** | Cysteine protease. Unclear biological function^1–766^. |
| **SUMF2** |  |
| **CLMP** |  |
| **CLUL1** |  |
| **KLK10** |  |
| **LRP11** |  |
| **MATN3** |  |
| **NOMO1** |  |
| **SERPINB6** |  |
| **SERPINB8** |  |
| **TINAGL1** |  |
| **NT-proBNP** | Natriuretic peptide. Unclear role in the CNS. |

**Supplemental table 8: Cellular origin of proteins correlated to KP activation**

| **Protein** | **Expressed by*** |
| --- | --- |
| CXCL9 | Macrophages |
| IL12B | Monocytes |
| TNFRSF9 | Granulocytes, Monocytes |
| TYMP | Monocytes |
| NADK | Monocytes |
| MSR1 | Macrophages |
| GZMA | NK-cells |
| CXCL10 | Macrophages |
| Flt3L | T-cells |
| IL6 | Fibroblasts |
| CTSC | Macrophages |
| CCL3 | Macrophages |
| CXCL11 | Macrophages |
| CRTAM | DC, T-cells |
| TRAIL | Granulocytes |
| ANGPTL1 | Fibroblasts |
| SIGLEC1 | Macrophages |
| METRNL | DC, Macrophages |
| CTSS | Monocytes, Macrophages |
| CD79B | B-cells |
| IL10B | Fibroblasts |
| Flt3L | T-cells |
| TNFSF14 | T-cells, DC |
| CCL11 | Fibroblasts |
| RNASE3 | Eosinophils |
| MCP1 | SMC, Fibroblasts, Macrophages |
| CLM1 | Macrophages |
| CD40 | Monocytes |
| CCL23 | Endothelialcells |

Data from the human protein atlas^67^

* Excluding cells from organs distant to the central nervous system.

DC: Dendritic Cells, SMC: Smooth Muscle Cells , KP: Kynurenine Pathway

**
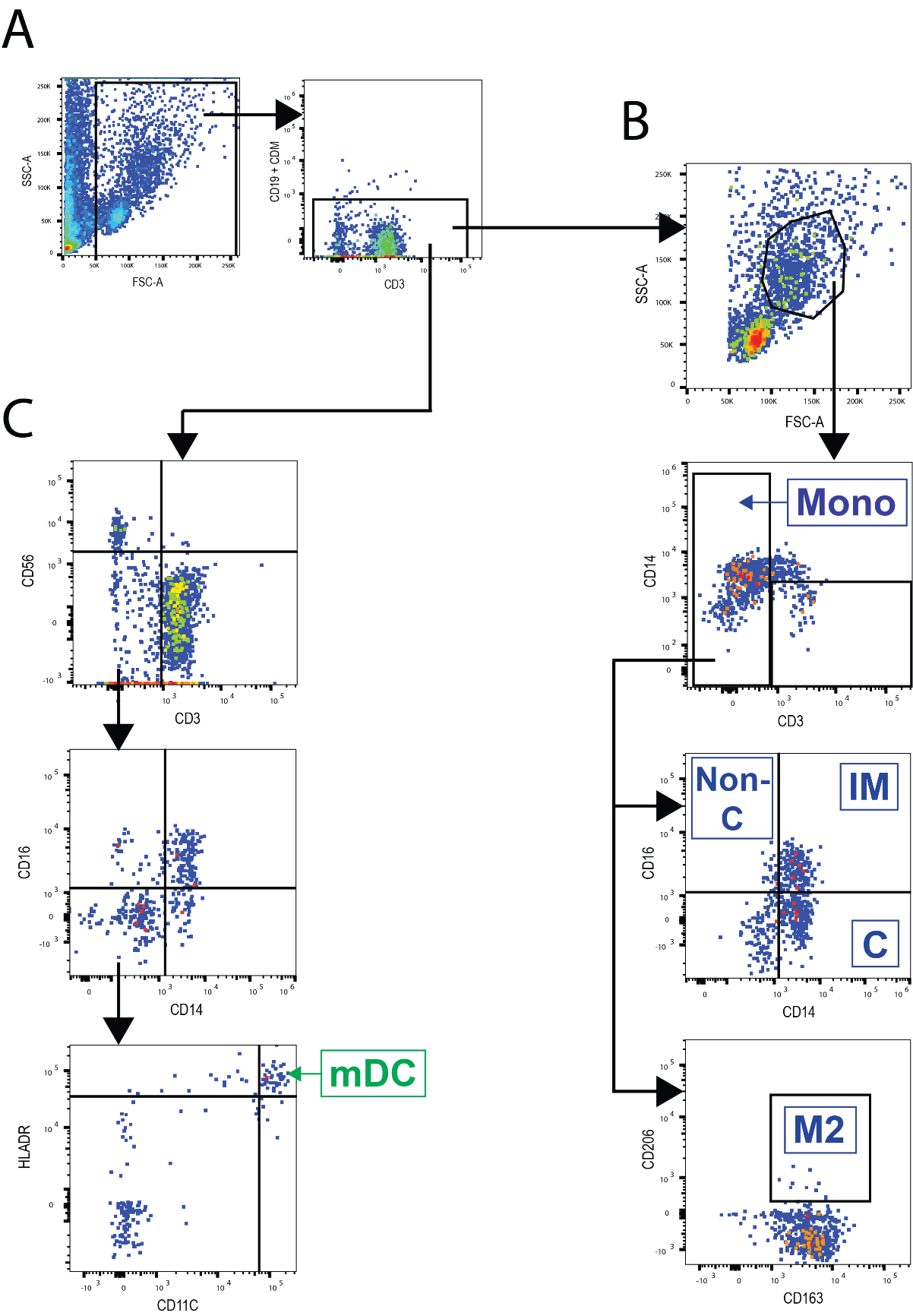
**

**Supplemental figure 1: Gating strategy for dendritic cells and monocytes.** After removal of debris, B-cells and dead cells **(A)**, monocytes were defined as FSChiSSChi and CD3- **(B)**. The monocytes were further divided into classical, non-classical and intermediate subsets, and M2 monocytes were defined as CD163+CD206+. mDCs were defined as CD3-CD56-CD16-CD14-HLADR+CD11c+ **(C)**. FSC: Forward Scatter, SSC: Side scatter, CDM: Cell Dead Marker, Mono: Monocytes, C: Classical, Non-C: Non-classical, IM: Intermediate, mDC: myeloid Dendritic Cells.

**
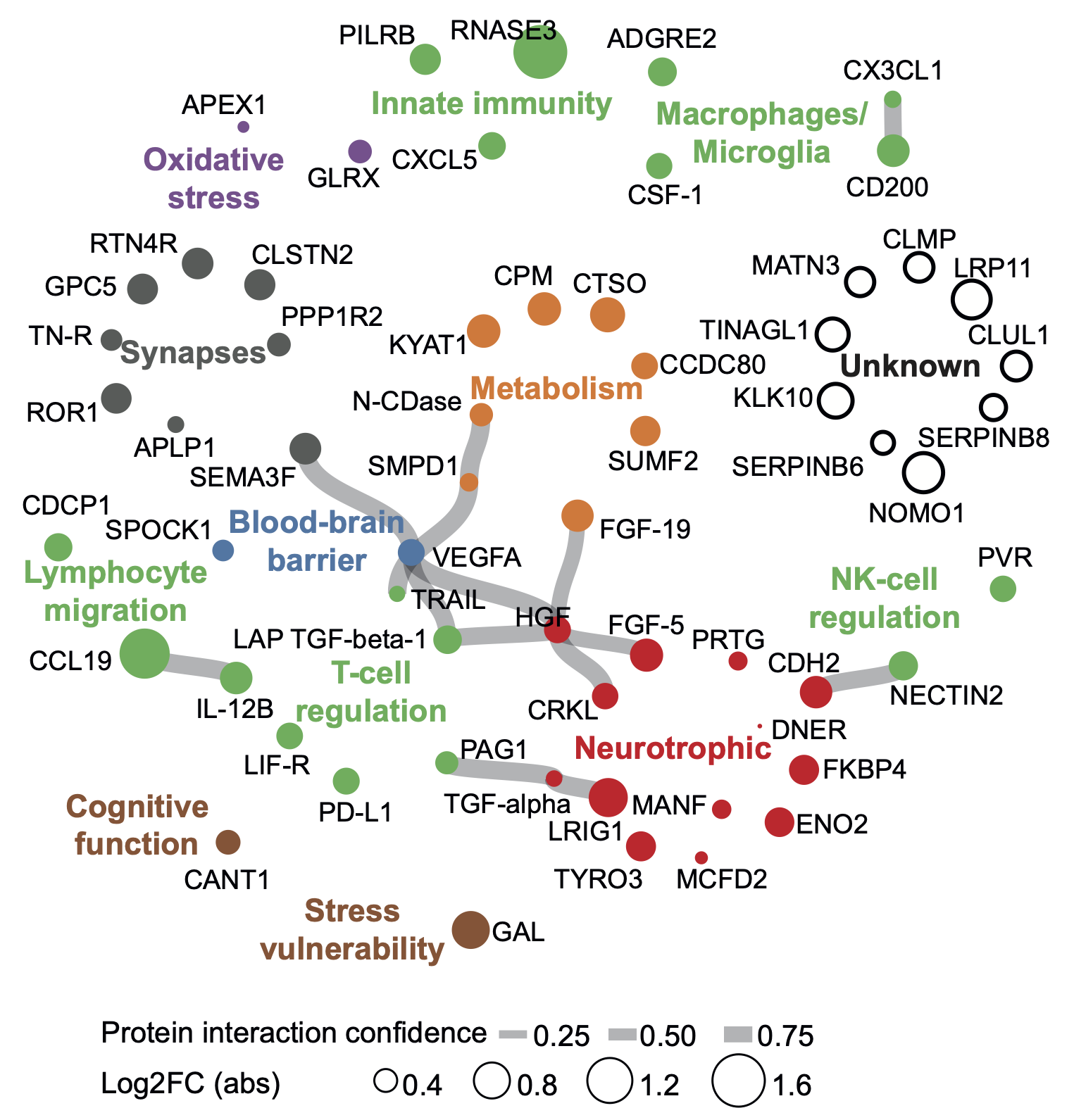
**

**Supplemental figure 2: Differentially expressed proteins grouped by function according to literature search.** The results of the literature search are demonstrated in supplemental table 4. Gray lines depict highly confident protein interactions according to a STRING database search.

**
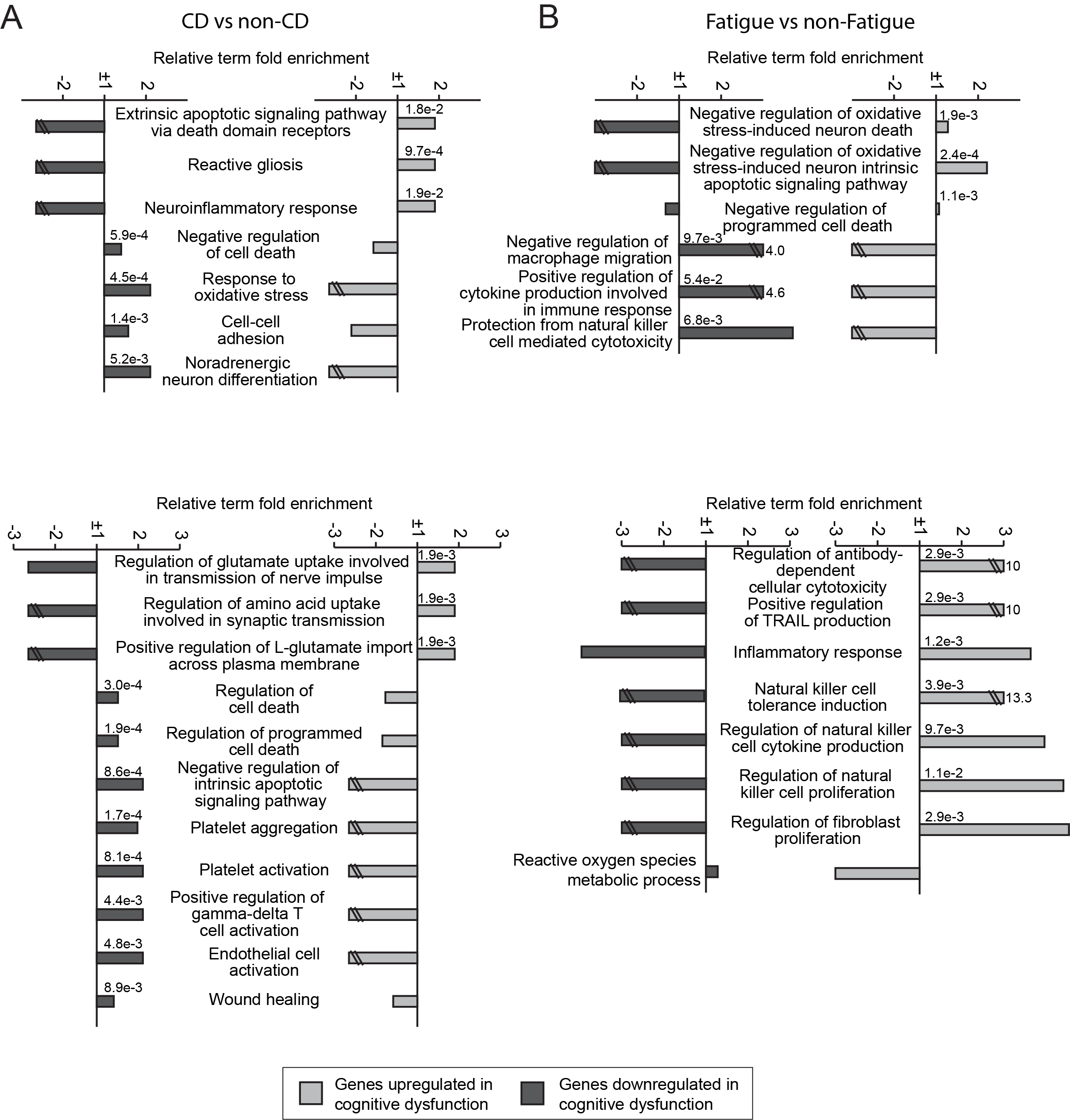
**

**Supplemental figure 3: Selected GO-terms based on mRNA sequencing analysis of extracellular vesicles**

All selected GO-terms from the results of the Panther Gene Ontology Analysis. The relative fold enrichment among both up- and downregulated mRNA are displayed. The terms that were enriched among the downregulated genes were negatively enriched among the upregulated genes and vice versa. The P-values prior to FDR adjustment are displayed. A) CD vs non-CD patients. B) Fatigued vs non-fatigued patients. GO: Gene Ontology, CD: Cognitive Dysfunction

**
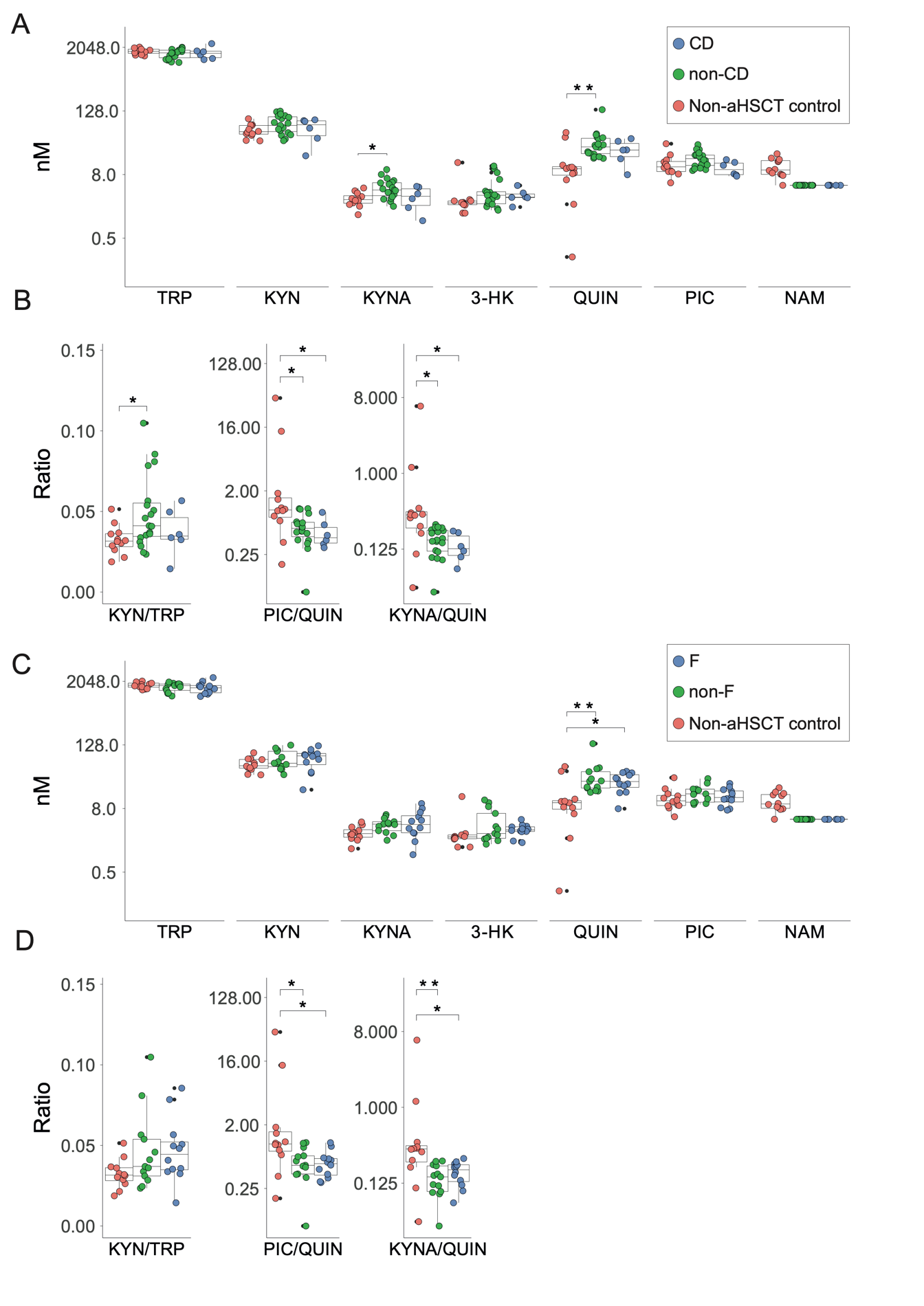
**

**Supplemental figure 4: Kynurenine pathway metabolites in CD vs non-CD and F vs non-F.** CD: Cognitive Dysfunction, F: Fatigue, NINDC: Non-inflammatory Neurological Disease Control.

**
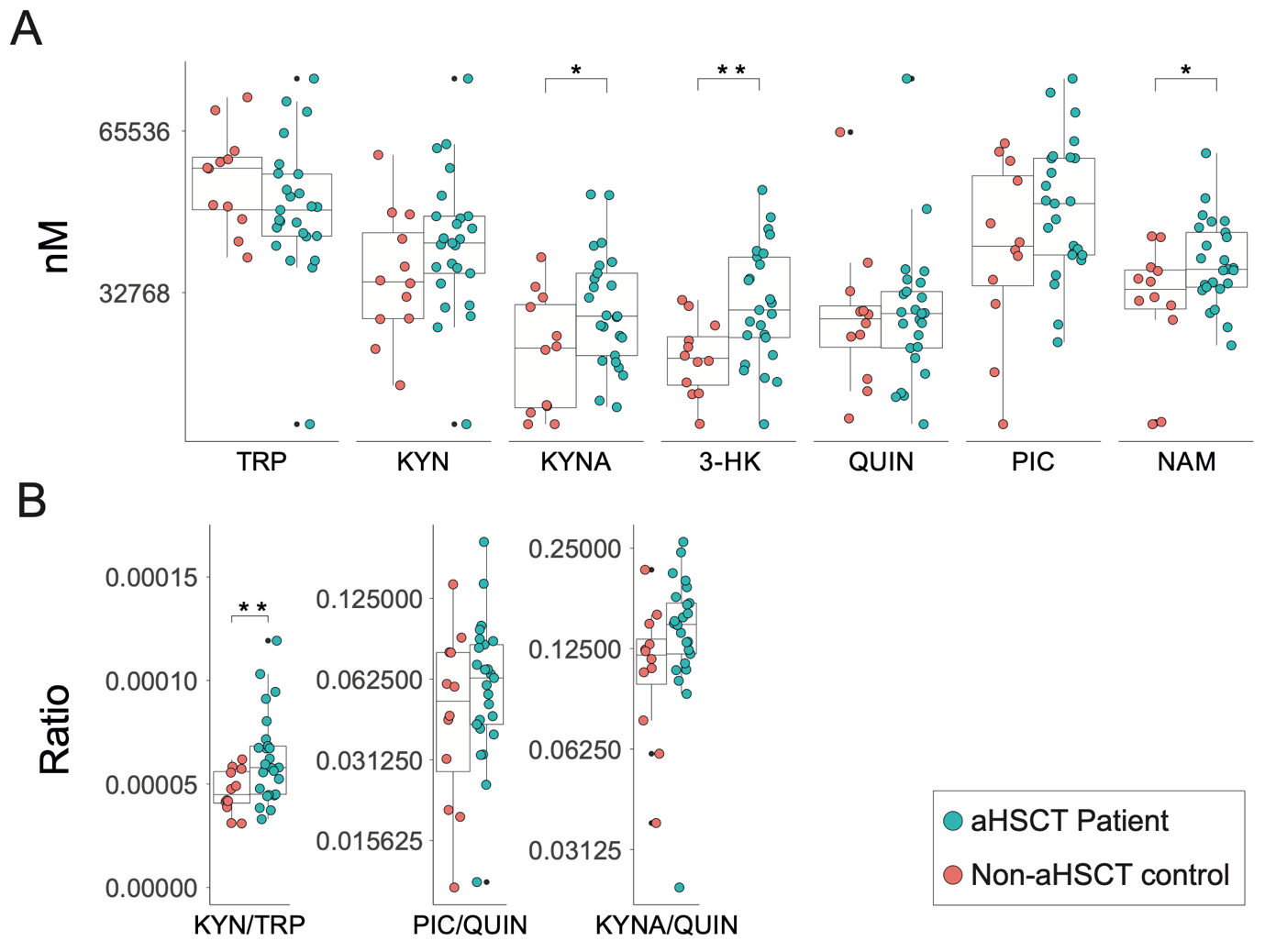
**

**Supplemental figure 5: Kynurenins in plasma, aHSCT patients vs NINDC.** NINDC: Non-inflammatory Neurological Disease Control.

**References**

1. Assarsson, E. *et al.* Homogenous 96-plex PEA immunoassay exhibiting high sensitivity, specificity, and excellent scalability. *PloS One* **9**, e95192 (2014).

2. Picelli, S. *et al.* Smart-seq2 for sensitive full-length transcriptome profiling in single cells. *Nat. Methods* **10**, 1096–1098 (2013).

3. Schwieler, L. *et al.* A novel, robust method for quantification of multiple kynurenine pathway metabolites in the cerebrospinal fluid. *Bioanalysis* **12**, 379–392 (2020).

4. Trepci, A. *et al.* Quantification of Plasma Kynurenine Metabolites Following One Bout of Sprint Interval Exercise. *Int. J. Tryptophan Res. IJTR* **13**, 1178646920978241 (2020).

5. O’Brown, N. M. *et al.* *The secreted neuronal signal Spock1 regulates the blood-brain barrier*. http://biorxiv.org/lookup/doi/10.1101/2021.10.13.464312 (2021) doi:10.1101/2021.10.13.464312.

6. Obermeier, B., Daneman, R. & Ransohoff, R. M. Development, maintenance and disruption of the blood-brain barrier. *Nat. Med.* **19**, 1584–1596 (2013).

7. Jin, K. *et al.* Vascular endothelial growth factor (VEGF) stimulates neurogenesis in vitro and in vivo. *Proc. Natl. Acad. Sci. U. S. A.* **99**, 11946–11950 (2002).

8. Faden, M., Al-Zahrani, F., Arafah, D. & Alkuraya, F. S. Mutation of CANT1 causes Desbuquois dysplasia. *Am. J. Med. Genet. A.* **152A**, 1157–1160 (2010).

9. Santos, I. *et al.* CXCL5-mediated recruitment of neutrophils into the peritoneal cavity of Gdf15-deficient mice protects against abdominal sepsis. *Proc. Natl. Acad. Sci. U. S. A.* **117**, 12281–12287 (2020).

10. Zhang, W. *et al.* CXCL5/CXCR2 axis in tumor microenvironment as potential diagnostic biomarker and therapeutic target. *Cancer Commun.* **40**, 69–80 (2020).

11. Banerjee, A. *et al.* Modulation of paired immunoglobulin-like type 2 receptor signaling alters the host response to Staphylococcus aureus-induced pneumonia. *Infect. Immun.* **78**, 1353–1363 (2010).

12. Bystrom, J., Amin, K. & Bishop-Bailey, D. Analysing the eosinophil cationic protein - a clue to the function of the eosinophil granulocyte. *Respir. Res.* **12**, 10 (2011).

13. Yan, Y. *et al.* CCL19 and CCR7 Expression, Signaling Pathways, and Adjuvant Functions in Viral Infection and Prevention. *Front. Cell Dev. Biol.* **7**, 212 (2019).

14. Krumbholz, M. *et al.* CCL19 is constitutively expressed in the CNS, up-regulated in neuroinflammation, active and also inactive multiple sclerosis lesions. *J. Neuroimmunol.* **190**, 72–79 (2007).

15. Enyindah-Asonye, G. *et al.* CD318 is a ligand for CD6. *Proc. Natl. Acad. Sci.* **114**, E6912–E6921 (2017).

16. Valente, T., Serratosa, J., Perpiñá, U., Saura, J. & Solà, C. Alterations in CD200-CD200R1 System during EAE Already Manifest at Presymptomatic Stages. *Front. Cell. Neurosci.* **11**, 129 (2017).

17. Pons, V. & Rivest, S. New Therapeutic Avenues of mCSF for Brain Diseases and Injuries. *Front. Cell. Neurosci.* **12**, 499 (2018).

18. Lauro, C., Catalano, M., Trettel, F. & Limatola, C. Fractalkine in the nervous system: neuroprotective or neurotoxic molecule? *Ann. N. Y. Acad. Sci.* **1351**, 141–148 (2015).

19. Chang, G.-W. *et al.* CD312, the human adhesion-GPCR EMR2, is differentially expressed during differentiation, maturation, and activation of myeloid cells. *Biochem. Biophys. Res. Commun.* **353**, 133–138 (2007).

20. Ushach, I. *et al.* Meteorin-like/Meteorin-β Is a Novel Immunoregulatory Cytokine Associated with Inflammation. *J. Immunol. Baltim. Md 1950* **201**, 3669–3676 (2018).

21. Huang, S. *et al.* The blooming intersection of subfatin and metabolic syndrome. *Rev. Cardiovasc. Med.* **22**, 799–805 (2021).

22. Parveen, F. *et al.* Role of Ceramidases in Sphingolipid Metabolism and Human Diseases. *Cells* **8**, E1573 (2019).

23. Savitz, J. The kynurenine pathway: a finger in every pie. *Mol. Psychiatry* **25**, 131–147 (2020).

24. Tremblay, F. *et al.* Loss of coiled-coil domain containing 80 negatively modulates glucose homeostasis in diet-induced obese mice. *Endocrinology* **153**, 4290–4303 (2012).

25. Lan, T. *et al.* FGF19, FGF21, and an FGFR1/β-Klotho-Activating Antibody Act on the Nervous System to Regulate Body Weight and Glycemia. *Cell Metab.* **26**, 709-718.e3 (2017).

26. Marcelin, G. *et al.* Central action of FGF19 reduces hypothalamic AGRP/NPY neuron activity and improves glucose metabolism. *Mol. Metab.* **3**, 19–28 (2014).

27. Mihaylova, V. *et al.* Highly variable neural involvement in sphingomyelinase-deficient Niemann–Pick disease caused by an ancestral Gypsy mutation. *Brain* **130**, 1050–1061 (2007).

28. Accogli, A. *et al.* De Novo Pathogenic Variants in N-cadherin Cause a Syndromic Neurodevelopmental Disorder with Corpus Callosum, Axon, Cardiac, Ocular, and Genital Defects. *Am. J. Hum. Genet.* **105**, 854–868 (2019).

29. Park, T.-J. & Curran, T. Crk and Crk-like play essential overlapping roles downstream of disabled-1 in the Reelin pathway. *J. Neurosci. Off. J. Soc. Neurosci.* **28**, 13551–13562 (2008).

30. Saito, S.-Y. & Takeshima, H. DNER as key molecule for cerebellar maturation. *Cerebellum Lond. Engl.* **5**, 227–231 (2006).

31. Haque, A., Polcyn, R., Matzelle, D. & Banik, N. L. New Insights into the Role of Neuron-Specific Enolase in Neuro-Inflammation, Neurodegeneration, and Neuroprotection. *Brain Sci.* **8**, E33 (2018).

32. Lindholm, D. *et al.* Fibroblast growth factor-5 promotes differentiation of cultured rat septal cholinergic and raphe serotonergic neurons: comparison with the effects of neurotrophins. *Eur. J. Neurosci.* **6**, 244–252 (1994).

33. Shim, S. *et al.* Peptidyl-prolyl isomerase FKBP52 controls chemotropic guidance of neuronal growth cones via regulation of TRPC1 channel opening. *Neuron* **64**, 471–483 (2009).

34. Maina, F. & Klein, R. Hepatocyte growth factor, a versatile signal for developing neurons. *Nat. Neurosci.* **2**, 213–217 (1999).

35. Benkhoucha, M. *et al.* Hepatocyte growth factor inhibits CNS autoimmunity by inducing tolerogenic dendritic cells and CD25+Foxp3+ regulatory T cells. *Proc. Natl. Acad. Sci.* **107**, 6424–6429 (2010).

36. Jeong, D. *et al.* LRIG1-Mediated Inhibition of EGF Receptor Signaling Regulates Neural Precursor Cell Proliferation in the Neocortex. *Cell Rep.* **33**, 108257 (2020).

37. Petrova, P. *et al.* MANF: a new mesencephalic, astrocyte-derived neurotrophic factor with selectivity for dopaminergic neurons. *J. Mol. Neurosci. MN* **20**, 173–188 (2003).

38. Toda, H. *et al.* Stem cell-derived neural stem/progenitor cell supporting factor is an autocrine/paracrine survival factor for adult neural stem/progenitor cells. *J. Biol. Chem.* **278**, 35491–35500 (2003).

39. Wong, Y.-H. *et al.* Protogenin defines a transition stage during embryonic neurogenesis and prevents precocious neuronal differentiation. *J. Neurosci. Off. J. Soc. Neurosci.* **30**, 4428–4439 (2010).

40. Junier, M. P. What role(s) for TGFalpha in the central nervous system? *Prog. Neurobiol.* **62**, 443–473 (2000).

41. Anchan, R. M., Reh, T. A., Angello, J., Balliet, A. & Walker, M. EGF and TGF-alpha stimulate retinal neuroepithelial cell proliferation in vitro. *Neuron* **6**, 923–936 (1991).

42. Akkermann, R. *et al.* The TAM receptor Tyro3 regulates myelination in the central nervous system. *Glia* **65**, 581–591 (2017).

43. Pende, D. *et al.* PVR (CD155) and Nectin-2 (CD112) as ligands of the human DNAM-1 (CD226) activating receptor: involvement in tumor cell lysis. *Mol. Immunol.* **42**, 463–469 (2005).

44. Chan, C. J. *et al.* The receptors CD96 and CD226 oppose each other in the regulation of natural killer cell functions. *Nat. Immunol.* **15**, 431–438 (2014).

45. Pei, D.-S., Jia, P.-P., Luo, J.-J., Liu, W. & Strauss, P. R. AP endonuclease 1 (Apex1) influences brain development linking oxidative stress and DNA repair. *Cell Death Dis.* **10**, 348 (2019).

46. Diwakar, L., Kenchappa, R. S., Annepu, J. & Ravindranath, V. Downregulation of glutaredoxin but not glutathione loss leads to mitochondrial dysfunction in female mice CNS: implications in excitotoxicity. *Neurochem. Int.* **51**, 37–46 (2007).

47. Juhasz, G. *et al.* Brain galanin system genes interact with life stresses in depression-related phenotypes. *Proc. Natl. Acad. Sci.* **111**, E1666–E1673 (2014).

48. Schilling, S. *et al.* APLP1 Is a Synaptic Cell Adhesion Molecule, Supporting Maintenance of Dendritic Spines and Basal Synaptic Transmission. *J. Neurosci. Off. J. Soc. Neurosci.* **37**, 5345–5365 (2017).

49. Ranneva, S. V., Maksimov, V. F., Korostyshevskaja, I. M. & Lipina, T. V. Lack of synaptic protein, calsyntenin-2, impairs morphology of synaptic complexes in mice. *Synap. N. Y. N* **74**, e22132 (2020).

50. Kamimura, K. & Maeda, N. Glypicans and Heparan Sulfate in Synaptic Development, Neural Plasticity, and Neurological Disorders. *Front. Neural Circuits* **15**, 2 (2021).

51. Yang, H. *et al.* Protein Phosphatase-1 Inhibitor-2 Is a Novel Memory Suppressor. *J. Neurosci. Off. J. Soc. Neurosci.* **35**, 15082–15087 (2015).

52. Paganoni, S., Bernstein, J. & Ferreira, A. Ror1-Ror2 complexes modulate synapse formation in hippocampal neurons. *Neuroscience* **165**, 1261–1274 (2010).

53. Wills, Z. P. *et al.* The nogo receptor family restricts synapse number in the developing hippocampus. *Neuron* **73**, 466–481 (2012).

54. Tran, T. S. *et al.* Secreted semaphorins control spine distribution and morphogenesis in the postnatal CNS. *Nature* **462**, 1065–1069 (2009).

55. Dankovich, T. M. *et al.* Extracellular matrix remodeling through endocytosis and resurfacing of Tenascin-R. *Nat. Commun.* **12**, 7129 (2021).

56. Freeman, G. J. *et al.* Engagement of the PD-1 immunoinhibitory receptor by a novel B7 family member leads to negative regulation of lymphocyte activation. *J. Exp. Med.* **192**, 1027–1034 (2000).

57. Pagenstecher, A. *et al.* Astrocyte-targeted expression of IL-12 induces active cellular immune responses in the central nervous system and modulates experimental allergic encephalomyelitis. *J. Immunol. Baltim. Md 1950* **164**, 4481–4492 (2000).

58. Zhu, Y. *et al.* Identification of CD112R as a novel checkpoint for human T cells. *J. Exp. Med.* **213**, 167–176 (2016).

59. Janssens, K. *et al.* Leukemia inhibitory factor tips the immune balance towards regulatory T cells in multiple sclerosis. *Brain. Behav. Immun.* **45**, 180–188 (2015).

60. Brdicka, T. *et al.* Phosphoprotein associated with glycosphingolipid-enriched microdomains (PAG), a novel ubiquitously expressed transmembrane adaptor protein, binds the protein tyrosine kinase csk and is involved in regulation of T cell activation. *J. Exp. Med.* **191**, 1591–1604 (2000).

61. Li, M. O. & Flavell, R. A. TGF-beta: a master of all T cell trades. *Cell* **134**, 392–404 (2008).

62. Kandasamy, M. *et al.* TGF-beta signalling in the adult neurogenic niche promotes stem cell quiescence as well as generation of new neurons. *J. Cell. Mol. Med.* **18**, 1444–1459 (2014).

63. Ikeda, T. *et al.* Dual effects of TRAIL in suppression of autoimmunity: the inhibition of Th1 cells and the promotion of regulatory T cells. *J. Immunol. Baltim. Md 1950* **185**, 5259–5267 (2010).

64. Tisato, V., Gonelli, A., Voltan, R., Secchiero, P. & Zauli, G. Clinical perspectives of TRAIL: insights into central nervous system disorders. *Cell. Mol. Life Sci.* **73**, 2017–2027 (2016).

65. Deiteren, K., Hendriks, D., Scharpé, S. & Lambeir, A. M. Carboxypeptidase M: Multiple alliances and unknown partners. *Clin. Chim. Acta Int. J. Clin. Chem.* **399**, 24–39 (2009).

66. Reif, M. M., Mach, L. & Oostenbrink, C. Molecular insight into propeptide-protein interactions in cathepsins L and O. *Biochemistry* **51**, 8636–8653 (2012).

67. Uhlén, M. *et al.* Tissue-based map of the human proteome. *Science* **347**, 1260419 (2015).
